# Efficacy of the nucleoside analog 4′-Fluorouridine against Nipah virus in the Syrian hamster model

**DOI:** 10.1371/journal.ppat.1014093

**Published:** 2026-04-03

**Authors:** Olivier Escaffre, Madison L. Pearson, Terry L. Juelich, Jennifer K. Smith, Lihong Zhang, Carolin M. Lieber, Dariia Vyshenska, Tetsuro Ikegami, Griffin D. Haas, Rebecca E. Krueger, Zachary Sticher, Alexander A. Kolykhalov, Michael G. Natchus, George R. Painter, Kendra N. Johnson, Alexander L. Greninger, Robert M. Cox, Richard K. Plemper, Benhur Lee, Alexander N. Freiberg

**Affiliations:** 1 Department of Pathology, University of Texas Medical Branch, Galveston, Texas, United States of America; 2 Institute for Translational Sciences, University of Texas Medical Branch, Galveston, Texas, United States of America; 3 Center for Translational Antiviral Research, Georgia State University Institute for Biomedical Sciences, Atlanta, Georgia, United States of America; 4 Department of Laboratory Medicine and Pathology, University of Washington School of Medicine, Seattle, Washington, United States of America; 5 Department of Microbiology, Icahn School of Medicine at Mount Sinai, New York, United States of America; 6 Emory Institute for Drug Development, Emory University, Atlanta, GeorgiaUnited States of America; 7 Department of Microbiology and Immunology, University of Texas Medical Branch, Galveston, Texas, United States of America; 8 Institute for Biomedical Sciences, Georgia State University, Atlanta, Georgia, United States of America; Wageningen Bioveterinary Research, KINGDOM OF THE NETHERLANDS

## Abstract

Nipah virus (NiV) is a zoonotic paramyxovirus with pandemic potential, for which no licensed vaccines or therapeutics for human use are available. The nucleoside analog 4′-fluorouridine (4′-FlU, EIDD-2749) has broad activity against RNA viruses including *in vitro* activity against the Malaysia and Bangladesh clades of NiV (NiV-M and NiV-B, respectively). Here, we report the pharmacokinetic profile of orally administered 4′-FlU in Syrian Golden hamsters and its efficacy against NiV-B. 4’-FlU was orally bioavailable and provided sustained exposure of its bioactive anabolite 4’-FlU 5’-triphosphate (4′-FlU-TP) in the brain. A 7-day treatment course after NiV infection delayed time to death. Extending treatment to 21–28 days reduced viremia, incidence of lung disease, lung lesion severity, inflammation, and mortality. Lesions, viral antigen, or viral RNA could be detected in some survivors, suggesting persistent infections. Sequence analysis of NiV populations showed nonsynonymous single-nucleotide variants (SNVs) in some brain specimens from 4′-FlU-treated animals. Amino acid changes occurred in the nucleocapsid, the polymerase, or the phosphoprotein. It is currently unclear whether they reduce viral susceptibility to treatment or impact virulence but caused a slight increase in 4′-FlU potency compared to the genetic parent virus *in vitro*. However, none of these SNVs increased viral fitness in human brain astrocytes. This study established proof-of-concept for efficacious oral treatment of lethal NiV infection with a small-molecule nucleoside analog inhibitor in a relevant animal model.

## Author summary

Nipah virus is a deadly virus for which there are currently no approved treatments or vaccines for human use. In our study, we tested an experimental drug called 4′-fluorouridine (4′-FlU), which is taken orally and has shown promise against a variety of other viruses. Using a hamster model relevant to Nipah virus infection, we found that 4′-FlU was absorbed into the body and reached the brain. When we treated infected animals for a week, it delayed fatal disease development. Extending the treatment to three to four weeks also increased the chance of survival, reduced the amount of circulating virus in blood and lungs, and decreased signs of inflammation. However, some animals still had detectable amounts of virus or virus-related changes in their tissues after treatment, suggesting that the infection might not be completely cleared in every case. Some mutations in the viral genome were found in brain specimens of survivors. Our findings provide early evidence that 4′-FlU could be a useful oral treatment for Nipah virus, support further research into its use as a potential antiviral drug for henipaviruses, and highlight the importance of monitoring for potential adaptation and resistance.

## Introduction

Nipah virus (NiV; family *Paramyxoviridae*, genus *Henipavirus*) is a Tier 1 select agent risk group 4 zoonotic and highly pathogenic virus that has been emerging in Southeast Asia since 1998 [1]. NiV is on the World Health Organization (WHO) priority diseases list due to its high pandemic potential and lack of approved vaccines and therapeutics [2]. In humans, the incubation period ranges from 4 to 14 days, followed by onset of severe clinical signs such as acute encephalitis and respiratory illness with case fatality rates ranging from 40% to 100% [3]. Survivors frequently exhibit post-acute sequelae including neurological complications with paralysis [4,5]. Only in rare cases were infections asymptomatic [6]. Outbreaks occur on an almost yearly basis with the most recent ones being documented in 2026 in India.

Currently, there is no licensed vaccine or therapeutic treatment approved for human use. However, several prophylaxis candidates are being evaluated at advanced development stages [7], including the Chimpanzee adenovirus vector expressing NiV glycoprotein (ChAdOx1 NipahB), the mRNA-1215 vaccine encoding NiV fusion- and glycoprotein, the soluble glycoprotein vaccine (HeV-sG-V), the recombinant VSV vaccine platform (rVSV-Nipah, PHV02), as well as the m102.4 monoclonal antibody [8]. Based on estimated cost of treatment per patient, small molecules are far superior to antibody therapeutics and comparable to vaccines [7,9]. In addition, small molecules promise an immediate effect of decreasing morbidity and mortality in early stages of an outbreak. Our program pursues broad-spectrum orally available antivirals, which may protect against pathogens across multiple viral families. Nucleoside analogues have revolutionized antiviral therapy, but none have yet reached clinical trials for the NiV indication. An exception is ribavirin, which was evaluated during the initial outbreak in 1998. However, results were conflicting between an open-label trial, compassionate use during sporadic outbreaks, and *in vivo* studies using relevant animal models to study henipavirus disease [8–12]. In animals, remdesivir only partially protected non-human primates from NiV infection after delayed treatment [13], but prevented death when administered earlier through the intravenous route [14]. Favipiravir, previously approved for use against pandemic influenza virus and COVID-19 in some countries, showed some efficacy in the hamster model against NiV [15,16]. *In vitro*, other candidates have been tested [17–20], including an orally available version of remdesivir.

Recently, the ribonucleoside analog 4′-fluorouridine (4′-FIU, EIDD-2749) demonstrated antiviral activity against respiratory syncytial virus (RSV), SARS-CoV-2, Influenza A viruses, and several enteroviruses, arenaviruses, and alphaviruses [21–26]. Treatment also prevented death from a lethal challenge in some infection models even when initiated late after onset of clinical signs [23,25,27]. Importantly, 4′-FIU is efficacious when administered orally, which is followed by rapid intracellular conversion to its bioactive 5’-triphosphate (4′-FlU-TP) anabolite. Incorporation into nascent viral RNA stalls the polymerase complex [21,23], interrupting the viral replication cycle. *In vitro,* 4’-FlU reportedly blocks NiV polymerase with active concentrations (EC_50_) of 1.8 to 3.6 μM in minigenome reporter assays [21,28], and 0.1 to 2.9 μM against infectious NiV based on reporter virus, qPCR, and plaque assay read outs [25,28–30].

Here, we demonstrate oral efficacy of 4′-FIU against the recombinant Nipah virus Bangladesh strain expressing Gaussia luciferase (rNiV-B Gluc) in a stringent lethal hamster challenge model using a once-daily dosing regimen. We show that a 28-day 4′-FIU treatment offers significant protection - as seen by at least 60% increased survival, no shedding and no viremia - with room for further improvement through optimized treatment protocols.

## Materials and methods

### Ethics statement

Animal experiments were approved by the Institutional Animal Care and Use Committee (IACUC) at Georgia State University under protocol A25015 and conducted at ABSL-1.

### Pharmacokinetics (PK)

Female 4- to 5-weeks-old Syrian Golden Hamsters were obtained from Charles River and drug administration was performed via oral gavage (1 ml per 100 g body weight). For the single-dose PK study, subjects (n = 8 per group) were dosed once with either 2 or 10 mg/kg of 4′-FIU (EIDD-2749, provided by the Emory Institute of Drug Development). Five consecutive daily doses were administered in the multi-dose PK study. Blood was collected at 8 time points (between 30 minutes to 24hours) after dosing in both studies and tissues (brain and lung) from 4 subjects per group were collected at 3 and 24 hours post dosing. Calibration curve ranges were established using plasma and tissue blanks and ranged from 10 to 10,000 ng/ml for 4’-FlU in plasma, and 50–20,000 ng/g of tissue for 4’-FlU-TP. Quality control samples (3 concentrations) were run before and after analyzing a sample set using plasma and tissue blanks. Uridine 5’-triphosphate (UTP) was monitored as a control for sample integrity in tissues. Measured levels of UTP were approximately 3-times lower in the single-dose hamster groups compared to the repeated-dose and vehicle-dosed hamster groups.

### Ethics for studies with infectious agents

Animal experiments involving infectious NiV were conducted at animal biosafety level 4 (ABSL-4) at The University of Texas Medical Branch under The Institutional Animal Care and Use Committee (IACUC) protocol 2205033 and performed in accredited facilities, ensuring compliance with the guidelines of the Association for Assessment and Accreditation of Laboratory Animal Care International (AAALAC).

### Virus and cells

A recombinant Nipah virus that is a molecular copy of the SPB200401066 isolate (accession no. AY988601) from the 2004 outbreak in Rajbari district in Bangladesh [31], and encoding *Gaussia* luciferase (Gluc) and enhanced GFP reporter genes, was generated (rNiV-B Gluc) using the same strategy as the recombinant Malaysia prototype strain previously rescued [32] and sequenced for identity confirmation. The rNiV-B Gluc was then propagated in Vero E6 cells (CRL1586, ATCC, Gaithersburg, Maryland) to make a stock and was titrated by plaque assay technique using Vero (CCL81, ATCC, Gaithersburg, Maryland) [32]. The rNiV-B Gluc harboring mutations identified in this study on N, P, or L gene were generated from rNiV-B Gluc backbone by site-directed mutagenesis through in-fusion cloning and rescued by reverse genetics as previously described [32]. Whole plasmid sequences were verified (Plasmidsaurus) prior rescue and virus stocks were made from the first passage in Vero cells. Wild-type NiV-B (SPB200401066 isolate, accession no. AY988601) was also used in this study and stock was similarly prepared. For *in vitro* antiviral testing, both wild-type and recombinant viruses were used, while only rNiV-B Gluc was utilized in *in vivo* studies. All infectious work was conducted at BSL-4 at UTMB.

### Assessing 4′-FIU efficacy *in vitro* by plaque reduction assay

Vero cells (CCL81, ATCC, Gaithersburg, Maryland) were cultured in Dulbecco’s Modified Eagle Medium (DMEM) supplemented with 10% fetal bovine serum (FBS) and seeded at 2.5 × 10^5^ cells/well in 12-well plate format the day prior to infection. Virus inoculation was done with 100μl of wild-type NiV-B for 1 hour at 37°C, after which 1 mL of overlay was added (1% methylcellulose: 2X MEM, containing 2% FBS). Note that virus dose was adjusted to yield approximately 40 plaques. Plates were then incubated for 2 days at 37°C in a 5% CO_2_ incubator to allow plaque formation. Cells were then fixed with 10% buffered formalin with crystal violet for 30 minutes to visualize plaques. 4′-FIU was added during infection and supplemented into the overlay when assessing drug efficacy. The percent plaque reduction was calculated using the virus control (no drug in overlay) as reference. The effective concentration required to reduce plaques by 50% (EC_50_) was determined by plotting percent inhibition against 4′-FIU concentrations.

### Assessing 4′-FIU efficacy *in vitro* through a luminescence reporter assay

Vero cells (CCL81, ATCC, Gaithersburg, Maryland) were cultured in Dulbecco’s Modified Eagle Medium (DMEM) supplemented with 10% fetal bovine serum (FBS) and seeded at 10 x10^3^ cells/well in a 96-well plate the day prior to infection. A low infective dose of rNiV-B Gluc (MOI 0.001) was then added along with 4′-FIU where appropriate (total 100μl) and plates were incubated for 2 days at 37°C in a 5% CO_2_ incubator. Luminescence was detected after adding 50μl of Pierce Gaussia Luciferase Glow substrate (Pierce, Rockford, Illinois) per reaction using a Cytation 7 reader (Agilent, Santa Clara, California) at BSL-4. Percentage replication was calculated using the virus control (no drug in medium, biological triplicates for S1 Fig) as reference. This assay was also used to concomitantly compare viral replication of parental rNiV-B Gluc and 3 rNiV-B Gluc harboring a single mutation on the N, P or L gene on distinct 96-well plates using biological quadruplicates (8 biological replicates per plates for each virus and mock controls). The effective concentration required to reduce the luminescence signal by 50% (EC_50_) was determined by plotting percent inhibition against 4′-FIU concentrations.

### Assessing *in vivo* efficacy of 4′-FIU in the Syrian hamster model for rNiV-B Gluc

Male and female 5- to 6-week-old Syrian Golden Hamsters (Envigo, Indianapolis, Indiana) were evenly divided into 2 cohorts of six groups each (n = 5 male and 5 female per group). Hamsters were anesthetized using isoflurane prior to each manipulation, including rNiV-B Gluc challenge, administration of 4′-FIU, or sampling (blood and swabs). A challenge dose of 10^5^ particle forming units (PFU) per animal was administered intraperitoneally (IP, 100μl per animal) on study day 0 where appropriate. Backtitration was performed to determine delivered infective dose. The 4′-FIU treatment was 10 or 25 mg/kg and administered once daily orally (PO, 100μl per animal) starting either immediately or 8–10 hours (Delayed) post-challenge. Animals were then dosed with 4′-FIU daily for a total of 7–28 days, depending on the cohort. Matching vehicle formulation was made with 10mM sodium citrate buffer, 0.5% Tween 80 and administered using the same method and treatment schedule in mock groups.

Two treatment cohorts were run, a Short treatment Protocol (n = 60) and an Extended treatment Protocol (n = 60). In cohort 1 study (short treatment protocol), two control (virus and mock) groups received the vehicle, with only the virus group challenged with virus. The remaining four infected groups received 10 or 25 mg/kg 4′-FIU for 7 consecutive days either immediately or 8–10 hours (delayed) post-challenge. Based on the results from this cohort, the dose of drug and duration of treatment was adjusted in the cohort 2 study (extended treatment protocol). Specifically, four groups received virus and 10 mg/kg 4′-FIU either immediately or 8–10 hours (delayed) post-challenge once daily but for a total of 28 days or for 21 consecutive days. The two last groups received the vehicle but only one was challenged with virus, similar to the cohort 1 study.

Hamsters were monitored for weight loss daily for the first 10 days or during peak disease, and then every 2^nd^ or 3^rd^ day until end of study. Weight change was calculated from baseline weight at day 0. Daily health checks were conducted until study termination (up to day 28 for cohort 1 study, and days 34 and 51 for cohort 2 study, respectively) for monitoring any clinical signs of disease. Note that animals were monitored for a minimum for 14 days after termination of treatment. Animals were scored 1 if appearing healthy, 2 if lethargic, 3 if scoring 2 plus exhibiting ruffled fur, hunched posture, orbital tightening, rapid or labored respiration, or 15% weight loss. Animals showing 20% or more weight loss, paralysis, ataxia, head tilt, or seizures (score of 4) were immediately euthanized, and terminal sampling (blood and tissues) was performed.

### Blood and swabs collection, and luminescence signal detection

Whole blood was collected in BD vacutainer serum collection tubes (BD, Franklin Lakes, New Jersey) to recover serum after centrifugation (8,000 g for 10 minutes). Oral and nasal swabs were collected using, respectively, Puritan PurFlock (REF 25–3317-U, Puritan Medical Products Company, Guilford, Maine) and Microbrush applicators (REF MUO400, Young innovations, Grafton, Wisconsin) and then stored in screw top vials containing 250ul PBS (Gibco, Grand Island, New York). Luminescence was detected with a Cytation 7 reader (Agilent, Santa Clara, California) using 20µl of sample and 50µl of Pierce Gaussia Luciferase Glow substrate (Pierce, Rockford, Illinois) per reaction in 96-well plates.

### Tissue processing and virus titration

Brain, lung, and liver tissues were collected after euthanasia and were homogenized in PBS (Gibco, Grand Island, New York) using a TissueLyser (Qiagen, Hilden, Germany) and stainless-steel beads, as previously described [33]. The liquid phase was recovered and then processed, along with terminal sera, for titration by plaque assay on Vero cells as previously described [33].

### Luminescence Reduction Neutralization Test (LRNT)

Terminal sera from extended treatment protocol cohort were heat inactivated at 56°C for 30min. Starting from a 5-fold dilution, sera were 7 times 2- and 3-fold serially diluted in microplate format using DMEM supplemented with 2% FBS. An infective dose (MOI of 0.0005) of rNiV-B Gluc was added (1:1 volume) where appropriate, and plates were incubated at 37°C for 1h under periodic agitation on plate shaker (400rpm). Sera-virus mixtures were then transferred into microplates pre-seeded with Vero cells (CCL81; 20,000 cells/well; DMEM with 2% FBS) and further incubated for 48h at 37°C in a 5% CO_2_ incubator. Luminescence signal was detected as described in the above section “Assessing 4′-FIU efficacy *in vitro* through a luminescence reporter assay”. Note that an automated reagent dispenser (ThermoFisher scientific, St. Louis, Missouri) was used to accurately dispense virus inoculum, Vero cells suspension, and Gluc substrate. Each sera from 4′-FIU-treated animals was evaluated in duplicates in distinct microplates. Each plate included virus (8 replicates), mock (4 replicates), and neutralization (4 replicates, mouse anti-Nipah G [nAH1.3], Absolute Antibody) controls. Values were normalized to virus and mock controls for each plate, and percentage replication was calculated. Curve fitting was done from plotting average percent inhibition against 4′-FIU concentrations and the effective concentration required to reduce the luminescence signal by 50% (LRNT_50_) was determined.

### Tissue histology

Brain, lung, and liver tissues were collected after euthanasia for histopathology ad immunohistochemistry (IHC) analyses. Briefly, tissues were first fixed in 10% neutral buffered formalin for 24 hours and for an additional 48 hours with fresh buffered formalin in a clean container in BSL-4 following approved protocols before removal and processing of specimens under BSL-2 conditions. Tissues were embedded with paraffin prior sectioning (5 µm thickness). Sections were stained with hematoxylin and eosin for histopathological analyses. Others were processed for detection of NiV antigen using an anti-nucleocapsid antibody provided by Dr. C. Broder (Uniformed Services University, Bethesda, Maryland) or commercially available (HL1436, ThermoFisher Scientific, St. Louis, Missouri), an HRP-conjugated secondary antibody (ab7090, Abcam, Eugene, Oregon), and chromogenic substrate (NovaRED, VectorLabs, Newark, California). Review of slides and grading of lesions were performed by a veterinary pathologist.

### Multiplex immunoassay

Sera received a gamma irradiation treatment (5Mrad dose) on dry ice to inactivate virus and were then handled at BSL-2. A Hamster Cytokine Panel 1 Luminex 9-plex (Ampersand Biosciences, Lake Clear, New York) was used to detect interleukin (IL)-10, IL-2, IL-4, IL-6, interferon (IFN)-γ, monocyte chemoattractant protein 1 (MCP-1), macrophage inflammatory proteins (MIP)-1α, tumor necrosis factor alpha (TNFα), and vascular endothelial growth factor (VEGF) in sera following manufacturer recommendations. Samples were then run on a Bio-Plex 200 system and concentration of each analyte in sera was determined by plotting data against the corresponding standard curve.

### Sequencing of rNiV-B Gluc genome from stock and tissues

Virus stock and tissue specimens were homogenized in respectively TRIzol LS or TRIzol Reagent (Invitrogen, Carlsbad, California), as previously described [33] and RNA was extracted using Direct-zol RNA Miniprep kits (Zymo Research, Irvine, California). Detection of virus genome by quantitative RT-PCR assay was then performed using QuantiFast RT-PCR mix (Qiagen, Germantown, Maryland) and a set of primers (IDT, San Jose, California) and probe (TIB MOLBIOL, Adelphia, New Jersey) targeting the P gene as previously described [34]. Virus-positive tissues, mock controls, and virus stock were then submitted for viral whole-genome sequencing. Libraries were generated by converting the extracted RNA to double-stranded cDNA, which was subsequently fragmented, end-repaired, indexed, purified, and amplified using QIAseq xHYB Microbial Hyb&Lib Kit A (Qiagen, Germantown, Maryland). Library fragment sizes were estimated using the TapeStation 4200 D1000 (Agilent, Santa Clara, California), and concentrations were measured by Qubit 4 Fluorometer with the Qubit dsDNA HS Assay Kit (Invitrogen, Carlsbad, California). Lung, mock-infected, and virus stock libraries were shotgun sequenced using pre-capture libraries on Illumina NextSeq 2000 using a 2x150bp read format. Due to the high cellular content of brain specimens, these libraries were sequenced after affinity enrichment of Nipah virus sequences by hybridization capture. Hybridization capture was performed using the QIAseq xHYB Microbial Hyb&Lib Kit A with a custom Qiagen hybridization panel containing probes synthesized based on Nipah virus Malaysia (AF212302) [35] and Bangladesh (AY988601) [31] containing GLuc-P2A-GFP reporters [32]. The enriched libraries were further amplified and purified via bead clean-up. Library fragment sizes were estimated as described above and sequenced on the same instruments as lung samples. Raw reads were trimmed and quality filtered using fastp (v0.23.4) [36]. The QC-filtered and trimmed reads from virus stock were randomly subsampled to 1 million pairs using seqtk (v1.4) (https://github.com/lh3/seqtk). The consensus sequence of virus stock was generated by majority voting using Geneious Prime with NiV-B genome [31] containing GLuc-P2A-GFP reporters. Variants were identified using RAVA workflow (https://github.com/greninger-lab/RAVA_Pipeline/tree/publications_2025_01_01) [36,37]. The virus stock consensus sequence was used as the reference for variant calling. Shotgun libraries were analyzed in paired-end mode, whereas capture libraries were analyzed in single-end mode. Raw sequencing data is publicly available in NCBI BioProject PRJNA1214874.

### Assessing viral fitness of rNiV-B Gluc viruses in the brain

Human primary astrocytes (HA, #1800, ScienCell, Carlsbad, California) were cultured using the recommended astrocyte medium and protocol, per manufacturer instructions. HA were seeded at 10^5^ cells/well in 24-well plate format the day prior to infection. Virus inoculation (MOI 0.01) was done with 100μl of inoculum for 1 hour at 37°C. Cells were rinsed with 200μl PBS twice prior adding 500μl of HA medium for the assay. Supernatants were collected and titrated by plaque assay as described above. Kinetic was performed with biological triplicates.

### Statistical analysis

Log-rank test was used for comparison of survival curves and Chi-square test was used to compare mortality rates. One-way ANOVA followed by Tukey’s multiple comparisons test was used to compare weight loss, luminescence signal, and immune response between groups. Kruskal-Wallis test followed by Dunn’s multiple comparisons test was used when data were not normally distributed. Point-biserial correlation was used to determine the relationship between weight or seroconversion level with survival outcome. Fisher’s exact test was used to compare frequency of clinical symptoms as well as infectious virus in sera or tissues between groups (* p < 0.05, ** p < 0.01, *** p < 0.001, **** p < 0.0001). Comparison of curves from rNiV-B Gluc datasets *in vitro* was done using the Extra sum-of-squares F test to test whether one curve fits for the replication of all viruses.

## Results

### *In vitro* efficacy of 4′-fluorouridine (4′-FlU, EIDD-2749) against NiV

4′-FlU ribonucleoside analog previously showed antiviral activity against NiV-M (SPB199901924 prototype isolate, and isogenic recombinant reporter NiVs) with EC_50_ ranging from 0.1 to 2.9 μM, depending on the assays applied [28,29]. To characterize the activity of 4′-FlU against a predominant pathogenic henipavirus genotype and validate antiviral activity prior to *in vivo* studies, we tested 4’-FlU against rNiV-B Gluc and its corresponding wild-type 2004 Bangladesh isolate (NiV-B). Virus (MOI 0.001) was added to cells along with 4′-FIU and plates were further incubated for 48h prior to luminescence acquisition or plaque count. 4′-FlU EC_50_ values were 2.00 and 3.66 μM for, respectively, rNiV-B Glucand NiV-B (**S1 Fig**), which was comparable to previous results and prompted testing of this drug candidate in animal studies.

### Pharmacokinetics studies in Syrian Golden hamster

**Fig 1:**
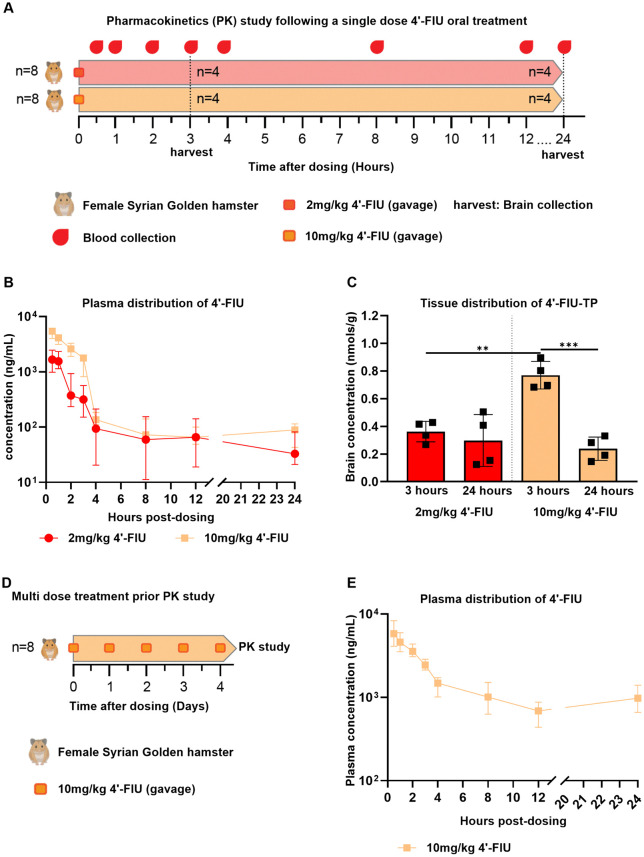
4’-Fluorouridine (4’-FIU) exposure in hamster following a single and multi-oral dose. (**A**) Timeline illustration of the pharmacokinetics study conducted after 4’-FIU oral gavage (single dose). Female hamsters (n = 8) received either 2 or 10 mg/kg 4’-FIU. Blood samples were collected as indicated prior final blood and tissue harvest at 24 hours post dosing. (**B**-**C**) 4’-FIU concentration in plasma and brain tissues over time. Note that the bioactive triphosphate form of 4’-Fluorouridine (4’-FIU-TP) is detected in the tissue. (**D**) Timeline illustration of the 4’-FIU oral gavage multi dose treatment prior to the pharmacokinetics study. Hamsters (n = 8) received 5 doses of 10 mg/kg 4’-FIU treatment prior serial blood collection as in illustration A. (**E**) 4’-FIU concentration in plasma over time. Data are presented as median with error or mean with standard deviation. Panel A and D partially created with BioRender. Plemper, R. (2026) https://BioRender.com/6ri40jk.

The pharmacokinetic properties of 4′-FlU have not previously been evaluated in hamsters. We administered 4′-FlU at 2 or 10 mg/kg of body weight to two groups of hamsters in an oral single-dose PK study (**Fig 1A**). Plasma concentrations of 4′-FlU were determined up to 24 hours post-dose (**Fig 1B**). 4’-FlU was rapidly absorbed following oral administration, reaching its maximum observed concentration (C_max_) by the time of first collection (30 minutes). There was a dose-dependent increase in C_max_ from 1,700–5,190 ng/ml between the 2 and 10 mg/kg groups, respectively. Concentration of free 4′-FlU in plasma then rapidly declined up to four hours post-dose, suggesting distribution to tissues. Plasma concentrations were relatively stable from 4 hours onwards with concentrations of 42 and 84 ng/ml at 24 hours post-dose in the 2 or 10 mg/kg group, respectively. Terminal half-lives were 16.6 and 35.4 hours for the 2 and 10 mg/kg groups, respectively. Brain tissue samples were collected at 3 and 24 hours post-dose to address whether 4’-FlU crosses the blood-brain barrier in Syrian golden hamsters and determine CNS exposure of its pharmacologically active metabolite, 4’-FlU-TP (**Fig 1C**). There was a dose-dependent increase in 4’-FlU-TP concentrations in the brain at three hours post-dose, indicating dose-dependency of drug tissue levels. The 10 mg/kg dose level was selected for a subsequent multi-dose PK study (**Fig 1D**). Plasma dynamics of 4’-FlU were similar after repeated dosing at 10 mg/kg/day compared to those after a single dose (**Fig 1E**). Plasma C_max_ reached 6,010 ng/mL after repeated dosing, but trough concentrations were approximately one order of magnitude higher, indicating that substantial accumulation occurred. Note that no evidence of 4’-FlU toxicity was noted during the pharmacokinetics studies.

### Proof-of-concept study of 7-day treatment with 4′-FlU against NiV (cohort 1 study: short treatment protocol)

**Fig 2:**
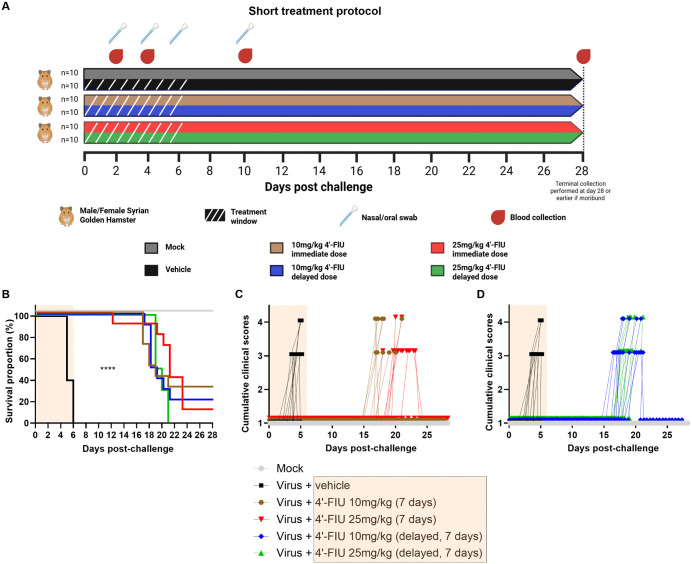
Efficacy of orally dosed 4’-FIU against an intraperitoneal rNiV-B Gluc challenge in the Syrian hamster model (Cohort 1). Five groups of hamsters (n = 10 per group, male and female evenly distributed) received each a targeted 10^5^ PFU dose of rNiV-B Gluc through the intraperitoneal route and were then either immediately or 8-10 hours later (delayed) treated daily and for 7 days with vehicle, or 10 to 25 mg/kg 4’-FIU. One additional group was used as negative control **(A)**. *Created in BioRender. Pearson,* ***M.*** *(2026)* https://BioRender.com/983h0ga. All six groups were further monitored for 21 days to assess for (B) survival as well as (**C, D**) clinical signs of disease. Note that some data sets are either (B) aligned or (C, D) staggered for improving visualization of values from each subject over time. The yellow shade (B, C, D, and in legend) indicates days of treatment. The Log-rank test was applied for comparison of survival curves and the Chi-square test was used to compare mortality rates. Asterisks ****) indicate statistical differences of p < 0.0001 between groups.

Considering its broad-spectrum activity against several members of the *Hareavirales* and *Mononegavirales* order [21,25,28,29,38], we first aimed to test anti-NiV efficacy when 4’-FlU was administered per oral once daily (q.d.) at 10 or 25 mg/kg for 7 days, starting either immediately post infection (pi) with a lethal rNiV-B Gluc inoculum or delayed 8–10 hpi (**Fig 2A**).

The short treatment protocol delayed disease onset: Five groups of hamsters (n = 10 per group, equal sex) were inoculated with a target dose of 10^5^ PFU/animal using the intraperitoneal (IP) route. Four groups received oral 4′-FlU treatment thereafter. Delivered dose was 2.5 × 10^4^ PFU and all subjects from the virus group (no treatment) succumbed to disease by day 6 (**Fig 2B**), which equated to a median survival of 5 days. Negligeable to mild weight loss occurred in 90% of animals prior to death (-7.9% range; -4.9% median) (**S2A Fig**) and was sometimes accompanied by respiratory and neurological signs of disease including labored breathing, paralysis, and tremor requiring euthanasia (**Fig 2C,2D**). Animals of the mock-infection group did not exhibit any signs of disease and survived until end of study (day 28) (**Fig 2B**) with an average body weight gain of 17% (**S2A Fig**). Treatment with 4′-FlU significantly (p < 0.0001) altered disease progression in all treatment arms and none of the animals in any treatment group died while receiving 4’-FlU. Time to death was increased by more than 13 days (median time between 18.5 to 21 days) compared to the virus control group (**Fig 2B–2D**). However, starting 4–8 days after treatment end, animals in the treatment group succumbed with fatality rates statistically indistinguishable from that of the vehicle control group (total fatality rate 34 of 40 animals, 85%, p = 0.19). Notably, excluding weight loss criteria, no sign of disease was observed up to day 12 in 4′-FlU-treated groups. After treatment end, clinical signs of NiV infection were identical in nature and severity in animals that had received 4′-FlU and succumbed to disease similar to those from the vehicle-treated control group (**Fig 2C,2D**), suggesting that 4′-FlU delayed disease onset. In addition, weight loss was generally greater and seen in more animals during the 7-day treatment period (p < 0.001 or 0.01, **S2B Fig**) and up to day 12 (**S2****C and** **S2D Fig**) when comparing the 25 versus 10 mg/kg cohorts or compared to the mock group, overall suggesting reduced tolerability of the higher dose. Inclusion of drug-only controls would be necessary to confirm this observation

**Fig 3:**
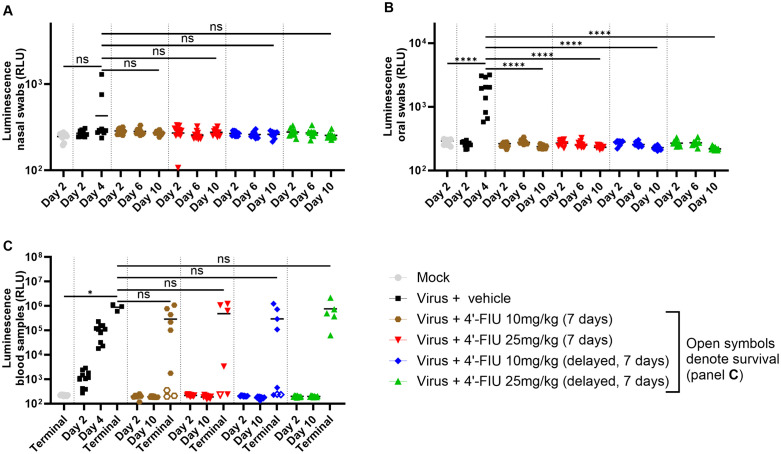
Efficacy of 4’-FIU at controlling rNiV-B Gluc shedding and dissemination in the Syrian hamster model (Cohort 1). **(A, B)** Nasal and oral shedding of virus was assessed during treatment (day 2, 4, and 6) and up to day 10 post challenge through detection of luminescence from the *Gaussia* luciferase activity. **(C)** Viremia was assessed through the same method as in A and **B.** Data are shown as mean with replicates in A, B, and **C.** Open symbols in C correspond to values from survivors from the challenge.

4′-FlU suppressed virus shedding and replication during treatment: Collection of nasal and oral swabs from each subject (**Fig 3A,3B**) allowed near real-time detection of virus replication during the first 10 days through monitoring luminescence intensity [32,39]. Consistent with the lack of clinical signs (excluding body weight loss) during that time frame, luciferase signals from swabs collected from 4′-FlU-treated hamsters did not increase and were comparable to the background signal of mock-infected animals, indicating efficient suppression of NiV replication. In contrast, at least 2 nasal (p > 0.05) and all oral (p < 0.0001) swabs from the vehicle control group showed elevated luciferase activity, concomitant with disease onset on day 4. Similar results were obtained from retro-orbital bleeds (**Fig 3C**), altogether revealing complete suppression of virus shedding and replication during treatment and at least up to 10 days post challenge. At study end, after treatment had been discontinued, reporter signals in sera from terminal bleeds of most (86%) animals that had received 4′-FlU and ultimately required euthanasia and from untreated animals were comparable (**Fig 3C**), indicating viremia.

Consistent with viremia, the proportion of animals that harbored infectious particles in lung and liver at some point in the study was similar across the different groups (**S2****E and** **S2F Fig**). However, virus emerged significantly later in 4’-FlU-treated animals, and the four treatment groups contained less subjects with virus in the brain (p > 0.05) compared to the vehicle group (**S2G Fig**), indicating that 4′-FlU interfered with viral host invasion.

### Proof-of-concept study of extended treatment with 4′-FlU against NiV (cohort 2 study)

Based on the observation that 4’-FIU prevented disease progression in the active treatment phase, we next asked whether a prolonged treatment course will facilitate viral clearance, resulting in full recovery of animals. Since we did not observe significant differences in efficacy between the different dose groups in the cohort 1 study, we selected only the 10 mg/kg dose level for this extended treatment course.

**Fig 4:**
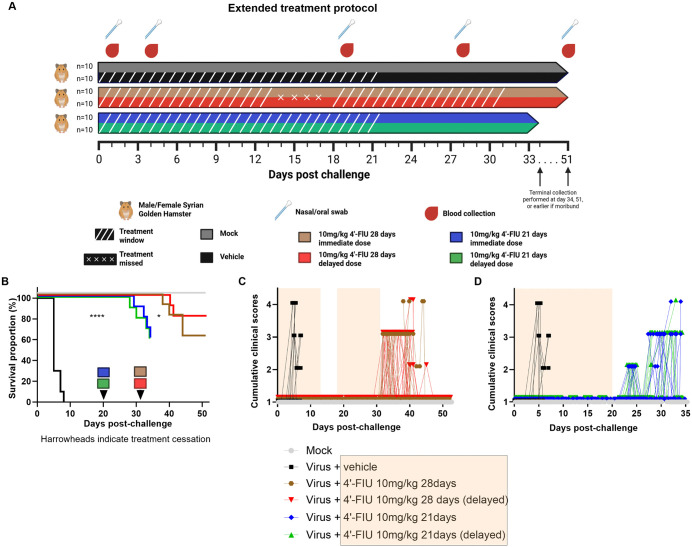
Effect of prolonged 4’-FIU treatment against an intraperitoneal rNiV-B Gluc challenge in the Syrian hamster model (Cohort 2). Four groups of hamsters (n = 10 per group, male and female evenly distributed) received each a targeted 10^5^ PFU dose of rNiV-B Gluc through the intraperitoneal route and were then dose similarly as in cohort 1 for 28 days total or 21 consecutive days with 10 mg/kg 4’-FIU. Two additional groups were used as virus and negative controls, similar to cohort 1 **(A)**. *Created in BioRender. Pearson,* ***M.*** *(2026)* https://BioRender.com/983h0ga. Animals were monitored up to day 34 or 51 after challenge to assess for (**B**) survival and (**C, D**) clinical signs of disease. Note that some data sets are either (B) aligned or (C, D) staggered for improving visualization of values from each subject over time. The yellow shade (C, D, and legend) indicates days of treatment whereas arrowheads (B) indicate treatment cessation. The Log-rank test was applied for comparison of survival curves, and the Chi-square test was used to compare mortality rates. Asterisks (*, ****) indicate statistical differences of respectively p < 0.05, and 0.0001 between groups.

The extended treatment courses improved survival: Five groups of hamsters were inoculated IP with a target dose of 10^5^ PFU per animal (actual dose delivered was 5.3 × 10^4^ PFU) and four groups received oral 4′-FlU treatment thereafter (**Fig 4A**). Compound was administered per oral for a total of 28 days with an unplanned 4-day gap at days 14–17, or for 21 consecutive days starting either immediately after infection or 8–10 hpi. A mock control group remained uninfected, and a reference group received vehicle only. All animals in the vehicle control group again succumbed rapidly to infection (median survival 5 days) (**Fig 4B**), showed rapid body weight loss (90% of animals) (**S3A Fig**), and developed lethargy progressing to respiratory and neurological endpoints (**Fig 4C**). Hamsters in the mock group remained healthy until end of study (day 51) and gained on average 36% from original weight (**S3A Fig**). Compared to the 7-day treatment regimen applied before, the extended treatment courses significantly improved survival (**Fig 4B**). When monitoring animals for an additional 20 days after completion of a 28-day treatment regimen, survival rates ranged from 60 to 80% compared to 0% in the virus control group (p < 0.01 or 0.001). Similarly, 60% survival was observed in animals monitored for 14 days after a 21-day continuous treatment course compared to 0% in virus control group (p < 0.01). Total study length for the two 21-day treatment groups was restricted to 34 days to probe for infectious particles in tissues of phenotypically healthy survivors (score of 1), or to monitor for potential delayed neurological symptoms. Disease progression varied notably between the different treatment groups (p < 0.05) (**Fig 4C,4D**). Mean time-to-death ranged from 40.5 to 42 days and from 31.5 to 32.5 days in groups receiving treatment for 28 or 21 days, respectively. All treated animals appeared healthy up to day 31 and 24 in these groups without body weight loss. These results indicate that both survival and delay in progression of disease are 4′-FlU treatment-dependent. Weight gain by the end of dosing in the 28-days treatment groups tended to be slightly delayed (p < 0.05) compared to mock groups (**S3B Fig**). Body weight on the last day of treatment and overall outcome were not correlated (point-biserial correlation; r ranging from -0.38 to 0.30). However, body weight gain of survivors between end of treatment and termination of study was steady in all groups and comparable to that of the mock group (**S3****A,** **S3****C, and** **S3D Fig**). Seroconversion to infection was assessed using samples collected at time of animal meeting criteria for euthanasia or end of study (**S3E Fig**). An LRNT_50_ higher than 10 (reciprocal dilution) could be calculated for most 4’-FlU-treated animals and tended to be lower (p < 0.01) in those from the 21- (range: 176, mean:43) versus 28- (range: 548, mean:138) day treatment regimen. However, seroconversion levels did not correlate with survival to infection (point-biserial correlation; r = -0.02).

**Fig 5:**
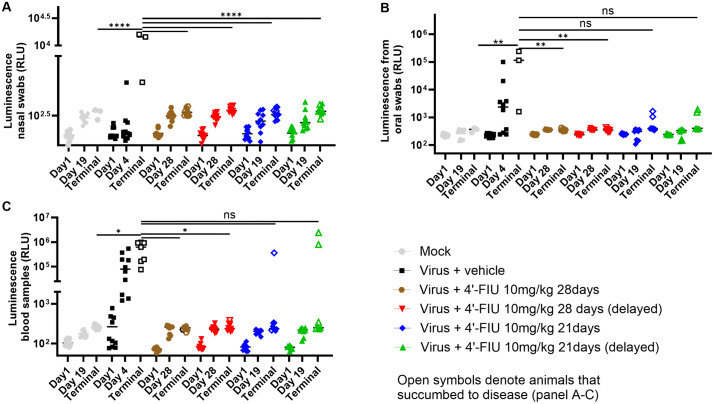
Effect of prolonged 4’-FIU treatment on rNiV-B Gluc shedding and dissemination in the Syrian hamster model (Cohort 2). **(A, B)** Nasal and oral shedding of virus was assessed during treatment (day 1, and 19 or 28) and at the end of study through detection of luminescence from the Gaussia luciferase activity. **(C)** Viremia was assessed through the same method as in A and **B.** Data are shown as mean with replicates in A, B, and **C.** Open symbols in A, B, and C correspond to values from subjects succumbing to disease. One way ANOVA followed by Tukey’s multiple comparisons test was used to compare shedding and viremia data between groups. Asterisks (*, **, ****) indicate statistical differences of respectively p < 0.05, 0.01, or 0.0001 between groups. “ns” for not significant.

The 28-day treatment course suppressed virus shedding, and viremia, as well as reduced incidence of respiratory disease: Luminescence signals in nasal swabs were elevated only in animals of the vehicle-treated control group at day 4 (10%) or when moribund (100%, p < 0.0001), whereas signals in all 4′-FlU-treated animals remained at background level, similar to those seen in mock-infected animals (**Fig 5A**). Luciferase activity in oral swabs from animals of the vehicle control group was up to two orders of magnitude higher than those in 4’-FlU-treated animals and the mock control group (p < 0.01) (**Fig 5B**). These data suggest that extended treatment with 4′-FlU efficiently suppressed virus shedding, especially in animals that received the compound for 28 days. Luminescence values in blood samples were high in all vehicle animals but remained at background level in the 4’-FlU cohorts except for three hamsters in the 21 days dosing groups, which showed high values at terminal sampling (**Fig 5C**). In line with this, infectious virus was retrieved in terminal blood of 86% of vehicle animals while it was undetectable in 95% of terminal samples from 4’-FlU-treated animals (**S4A Fig**). These results were also consistent with our observation that fewer subjects in the 4′-FlU-treated versus vehicle groups contained infectious virus in brain (approaching significance in 2 groups), lung (p < 0.05 to 0.0001) and liver (p < 0.01 to 0.001) tissues (**S4****B,** **S4****C, and** **S4D Fig**). Although the incidence of neurological symptoms was similar between groups, fewer respiratory clinical signs were present in groups receiving 4′-FlU for 28 (p < 0.0001) or 21 days (p > 0.05) (**S1 Table**), suggesting that prolonged treatment, especially the 28-day regimen, reduced viremia and NiV pneumonia in the hamster model.

**Fig 6:**
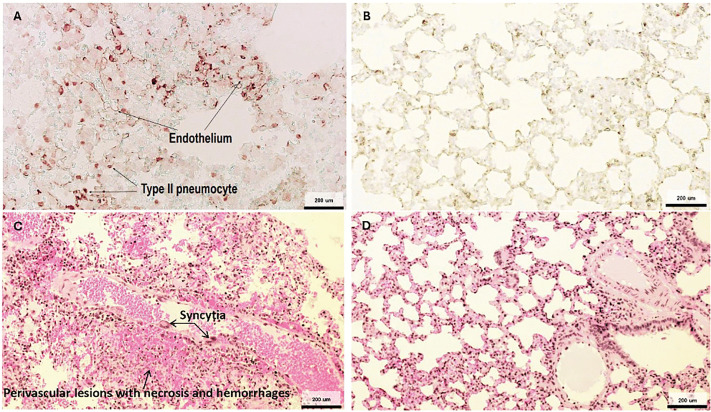
Virus nucleocapsid antigen distribution in the lung and associated lesions following rNiV-B Gluc challenge. **(A)** Virus nucleocapsid staining (dark red signal) in the alveolar area including in type II pneumocytes and endothelial cells of the small vasculature. **(B)** No specific virus nucleocapsid staining is observed in the alveolar space of mock tissues. **(C)** Hematoxylin and eosin staining showing virus-induced lung lesions including syncytia formation, necrosis, and hemorrhages. **(D)** Hematoxylin and eosin staining of a mock-infected untreated animal showing no remarkable lung lesions.

The 28-day treatment course reduced NiV-induced lung tissue pathology: Lung lobes from seven animals in the vehicle group showed NiV antigen in type II pneumocytes, and syncytia were frequently noted in endothelial cells of the pulmonary vasculature (**Fig 6A and** **S2 Table**), as opposed to lung sections derived from mock-infected hamsters (**Fig 6B**).

Histopathological analysis revealed that all subjects of the vehicle control group experienced either infiltration of mononuclear cells or developed necrotic and hemorrhagic lesions in the perivascular region and interstitium (**Fig 6C and** **S2 Table**). Mock-infected animals remained histopathologically unremarkable (**Fig 6D**). Lung edema or fibrinous exudate were furthermore consistently noted in animals in the vehicle control group (**S2 Table**). In contrast, lungs of 65% of animals in the 28-day 4′-FlU treatment groups were unremarkable, 30% showed mild interstitial increase of inflammatory cells, and only 5% presented with edema (**S2 Table**). Lung histopathology of 21-day treated animals did not fully correlate with disease outcome. Lesions were more frequent (68%) and presented predominantly more advanced (nodules, necrosis and edema) in hamsters with additional clinical signs (**S2 Table**), underscoring that a longer treatment course reduced NiV-induced lung pathology.

**Fig 7:**
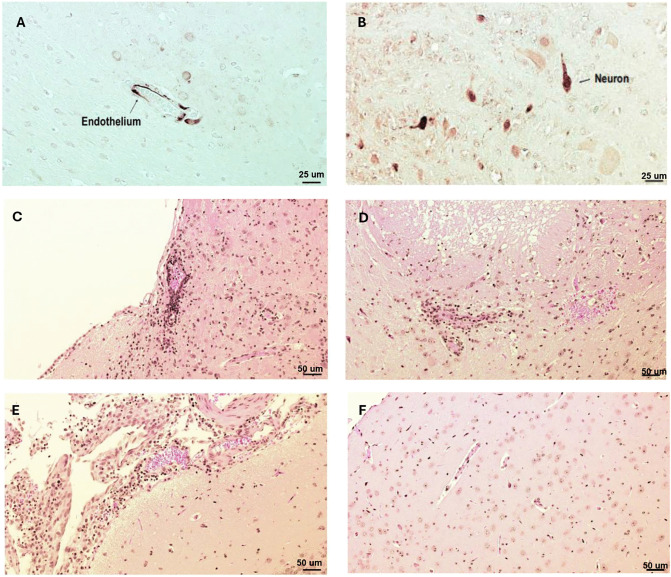
Virus nucleocapsid antigen distribution in the brain and associated lesions following rNiV-B Gluc challenge. **(A, B)** Virus nucleocapsid staining (dark red signal) in the hypothalamus, thalamus, and pons including in neurons, and endothelial cells. Hematoxylin and eosin staining showing virus-induced brain lesions including (**C**) mononuclear cells infiltrations, (**D**) focal necrosis, and (**E**) meningitis in the cortex. **(F)** Hematoxylin and eosin staining of a negative control showing no remarkable brain lesions.

Brain lesions did not correlate with neurological clinical signs or with presence of viral genome: Brain lesions in the vehicle group could only be detected based on focal demyelination in the thalamus of two subjects (28%) (**S2 Table**), including one animal presenting with paralysis, despite the presence of NiV antigen in endothelial cells and neurons in the striatum and hypothalamus (**Fig 7A,7B and** **S2 Table**). These animals also lacked neurological symptoms. Brain lesions were reported in 30% and 40% of, respectively, the 28- and 21-day 4′-FlU treatment groups and included mild to moderate increases in mononuclear cells in the perivascular region in pons (**Fig 7C and** **S2 Table**), focal necrosis in the thalamus (**Fig 7D and** **S2 Table**), mild to moderate meningitis in the cortex (**Fig 7E and** **S2 Table**), and focal demyelination in the thalamus (**S2 Table**). Brain histopathology did not consistently correlate with neurological signs including paralysis, tremor, and head tilt. No remarkable lesions were noted in the corresponding sections of mock-infected animals (**Fig 7F and** **S2 Table**). Viral genomic RNA could be retrieved from one brain of a survivor (28-day treatment group, **S2 Table**) that presented without neurological signs or brain lesions.

The 28-day treatment course prevented NiV-induced systemic inflammation: Circulating cytokines were quantified at the time of euthanasia, since they contribute to NiV pathogenesis [40,41] and can indicate changes in blood brain barrier (BBB) integrity in hamsters [42]. A 9-plex panel for IL (interleukin)-2, IL-4, IL-6, IL-10, Interferon (IFN)-γ, MCP-1, MIP-1α, TNFα & VEGF was tested with samples of the mock, vehicle, and pooled 28-day 4′-FlU treatment groups (**S5 Fig**). IFN-γ was significantly increased in hamsters in the vehicle group at peak disease compared to mock-infected and 4′-FIU-treated animals (p < 0.0001). Elevated IL-10 was present in the vehicle group compared to 4′-FIU-treated animals (p < 0.05), but not the mock group (p > 0.05). These results are consistent with the absence of pronounced lung lesions and viremia in animals treated for 28 days, indicating that treated and mock-infected animals did not develop systemic inflammation.

rNiV-B Gluc did not require adaptation to replicate in the lung of hamsters: To assess genetic integrity of compound-experienced virus populations, the rNiV-B Gluc genome of the virus stock was first deep sequenced and compared to reference sequence (human isolate, AY988601, [31]), which was appropriately modified to reflect the insertion of reporter genes between the nucleocapsid and phosphoprotein viral genes. Three single-nucleotide polymorphisms (SNP) with allele frequency (AF) over 99% were identified in the whole genome of rNiV-B Gluc, which corresponded to two nonsynonymous mutations in the fusion protein at amino acids (aa) 207 and 252 (**S3 Table**). No other SNPs with AF higher than 25% were detected. Then, NiV genome-positive tissues from the second cohort were used for sequencing and the same two nonsynonymous mutations in the fusion protein were found in infected tissues. Note that no coverage for NiV genome was found in mock-infected samples, confirming that these animals had not experienced virus. Of 11 lung tissue samples positive for virus RNA, 6 samples from the vehicle group and 3 samples from the 21-day 4′-FIU-treated group provided sufficient reads and coverage for reliable interpretations (**S2** **and** **S4 Tables**). No nonsynonymous or complex mutations with AF higher than 10% were found in the 3 lung samples of the 21-day 4′-FIU-treated animals compared to genomes recovered from the vehicle group (**S6 Fig**), and even a lower AF threshold (5%) did not reveal any changes in the RNA-dependent RNA-polymerase (RdRp) gene (L).

**Fig 8:**
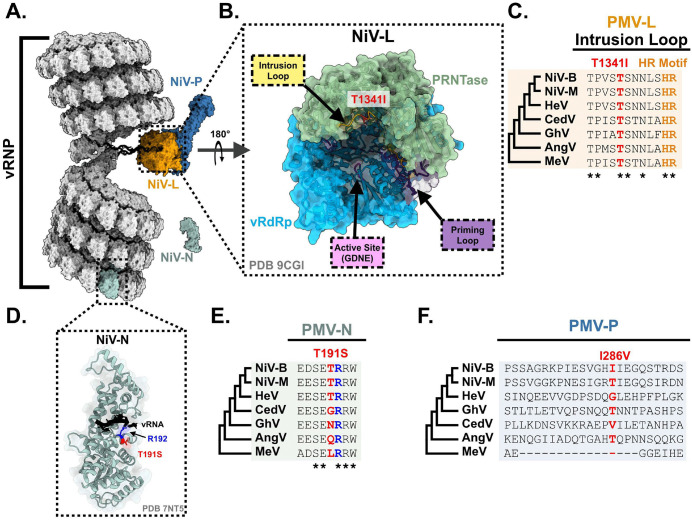
Nonsynonymous single-nucleotide variations are in components of the viral replicase. **(A)** Structural modeling depicting the viral replicase complex, consisting of the vRNA wrapped in N forming the vRNP, and displaced N where the RdRp, L, is threaded along the template vRNA; L is associated with a tetramer of **P.** PDB structures 7NT5 (NiV-N bound to RNA) and 9CGI (NiV-L and NiV-P) were manipulated in UCSF ChimeraX to generate these visualizations. NiV-N is depicted in light green, vRNA is depicted in black, NiV-P is depicted in blue, and NiV-L is depicted in yellow. **(B)** Visualization of the internal cavity of NiV-L with regions corresponding to the GDNE motif, priming loop, and intrusion loop indicated in pink, purple, and yellow, respectively. The T1341 residue identified in one virus as T1341I is highlighted in red. The PRNTase domain is depicted in green, whereas the vRdRp domain is depicted in light blue. **(C)** Amino acid alignment of the region of the intrusion loop from L proteins of NiV-B, NiV-M, HeV (Hendra virus), CedV (Cedar virus), GhV (Ghana virus), AngV (Angavokely virus), and MeV (Measles virus); T1341 and corresponding residues are highlighted in red, and the highly conserved HR motif is highlighted in orange. **(D)** Structural depiction of NiV-N bound to vRNA, with the T191 residue highlighted in red. Adjacent residue R192, which is involved in RNA binding, is shown in blue. **(E)** Amino acid alignment of the region of N containing T191 from NiV-B, NiV-M, HeV, CedV, GhV, AngV, and MeV. T191 and corresponding residues are highlighted in red. **(F)** Amino acid alignment of the region of P containing I286 from NiV-B, NiV-M, HeV, CedV, GhV, AngV, and MeV. I286 and corresponding residues are highlighted in red. For all sequence alignments, ClustalW was implemented in MEGA-X; phylogenetic trees depict topology only and were constructed for each alignment using the neighbor-joining method in MEGA-X. Conserved residues are shown with an * below each respective alignment. Numbering and mutants for each alignment refer to NiV Bangladesh sequences for each respective protein.

**Fig 9:**
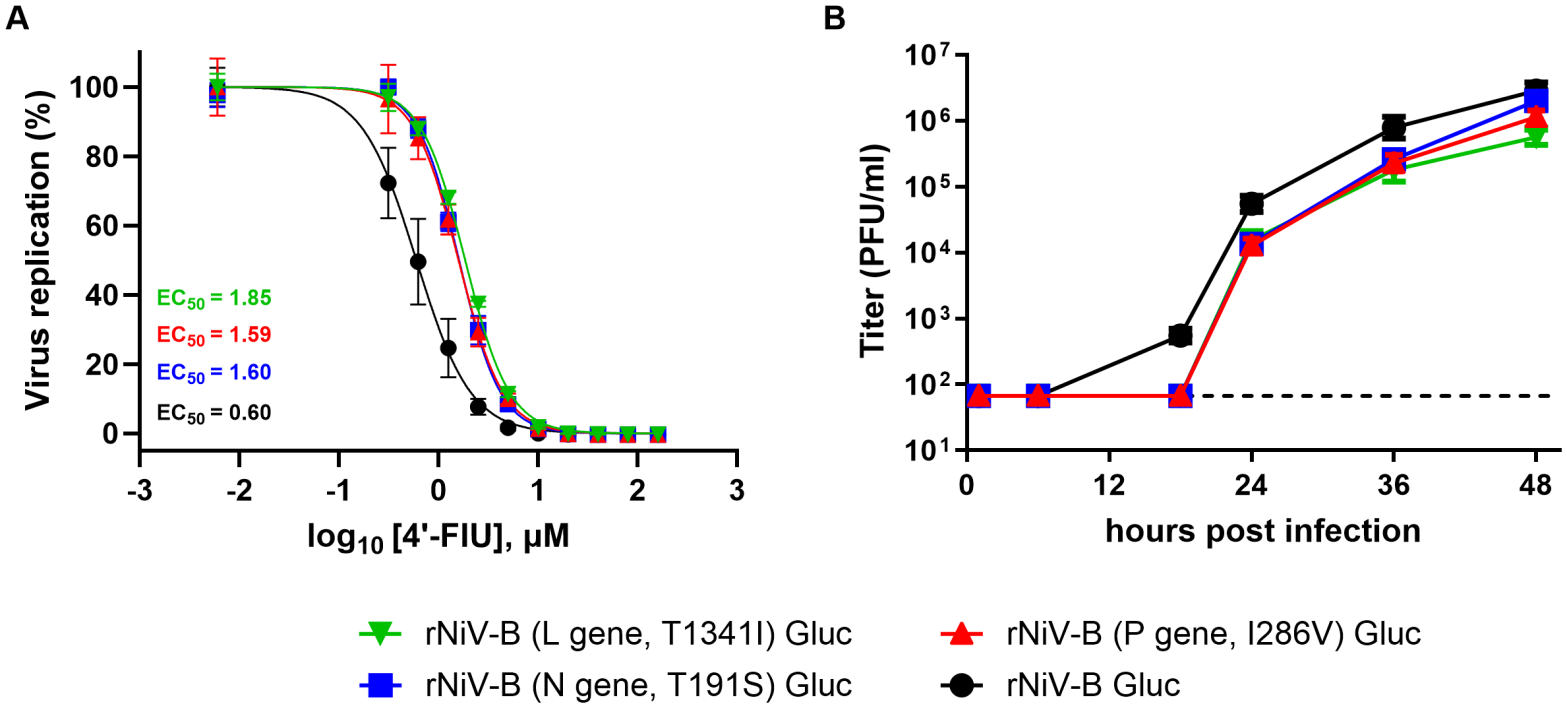
*In vitro* efficacy of 4’-FIU against rNiV-B Gluc incorporating T191S (N gene), I286V (P gene), or T1341I (L gene) change and viral fitness in human astrocytes. **(A)** Antiviral assays were concomitantly conducted in Vero CCL81 and on 4 individual 96-well plates with each mock and virus control (8 biological replicates). Four biological replicates were used per 4’-FIU concentration per plate. Determination of half-maximal effective concentration (EC_50_) against each virus at day 2 post-infection was done using the luminescence reporter on a Cytation 5 (Agilent). Values indicate the percentage of virus replication based on intensity of reporter gene expression from 4’-FIU- versus vehicle-treated infected cells. Results are expressed as the average of biological replicates, and error bars represent standard deviations. **(B)** Growth kinetic of rNiV-B Gluc and mutants in primary human astrocytes. Supernatants were collected 6 times between 1 and 48 hours post infection and were then tittered by plaque assay. Results are expressed as the average of biological triplicates, and error bars represent standard deviations. Horizontal dotted line shows the limit of detection.

rNiV-B Gluc did not require major adaptation to enter the CNS and 4′-FIU pressure induced adaptative changes to NiV replication complex: Only one SNV with AF > 25% was recovered from one of the seven sequenced untreated NiV-infected brain specimens (Matrix, G122*, 29%), suggesting that CNS-invasion does not require major NiV evolution in Syrian golden hamsters. Sequence analysis performed on the NiV genome-positive brain samples across the 21 (n = 6), and the 28-day (n = 5) 4′-FIU-treated groups (**S4 Table**) showed five nonsynonymous and nonsense single-nucleotide variants (SNV) with AF higher than 25% that were solely found in viral sequences from four hamsters that had received 4′-FIU (**S5 Table**). However, the corresponding lung tissues of these animals were negative for viral RNA and it could, therefore, not be determined whether these changes represent adaptation to the specific tissues or emerged in response to exposure to 4’-FlU. Three of the five SNVs were nonsynonymous, carried AF values higher than 96%, and were reported in three distinct samples from animals that succumbed to disease or met euthanasia criteria. Changes occurred either in the PRNTase domain of the RdRp (L gene, residue T1341I) [43] **(Fig 8A–8C)**, in the nucleocapsid (N) gene (residue T191S) near a region directly interacting with vRNA [44] **(Fig 8D–8E)**, or in the N-terminal domain of the phosphoprotein (P) gene (residue I286V) [43,45] **(Fig 8F).** The threonine at position 1341 of L was the most conserved of the 3 reported SNVs among henipaviruses (**Fig 8C**). A fourth specimen had two linked nonsense mutations in the P gene (E162*, E171*) at 53% and 38% AF, respectively. Due to lack of statistical power (n = 1 for each of these mutations), we inserted the 3 nonsynonymous SNVs individually into the rNiV-B Gluc backbone to assess whether either of these substitutions may represent a compound-induced change. Susceptibility of the recovered rNiV-B Gluc mutants to 4’-FlU was compared to that of the genetic parent virus *in vitro* (**Fig 9A**). Inhibitory concentrations were slightly increased by 2.5- to 3-fold compared to parental rNiV-B Gluc (EC_50_ ranging from 1.59 µM to 1.85 µM versus 0.6 μM, F test, p < 0.0001), altogether suggesting that these SNVs could provide a minor advantage to the virus population in the presence of 4’-FlU inhibitory pressure.

### The viral fitness of rNiV-B Gluc mutants is reduced compared to parental virus in human brain astrocytes

Viral genome and proteins have been detected in the CNS, including in astrocytes, across several animal models of Nipah virus disease [46–49] and these glial cells may also be affected in humans [50]. To evaluate the fitness of rNiV-B Gluc mutants in the brain, a 48-hour kinetic was performed using primary human astrocytes derived from the cerebral cortex (**Fig 9B**). The titers from rNiV-B Gluc mutants in the supernatants remained comparable from one another through the time course and tended to be slightly lower compared to the parent virus from 16 to 36 hours post infection (Extra Sum-of-Squares F test; p = 0.0004). Specifically, only the rNiV-B Gluc titer increased from below the limit of detection (LOD 67pfu/ml) to an average of 5.6 x 10^2^ pfu/ml by the 16-hour mark suggesting a minimum of about 10-fold difference compared to rNiV-B Gluc mutants. This slight advantage was still evident at 36 hours, and by the final time point, titers from the parent virus and two out of the three rNiV-B Gluc mutants exceeded 10^6^ pfu/ml. Together, these results indicate that human astrocytes are permissive to NiV and that the nonsynonymous SNVs in L, N, and P gene did not improve growth kinetics or increased maximal titers *in vitro*.

## Discussion

In search of novel, safe and efficacious therapeutics for the treatment of NiV disease, we demonstrate *in vivo* efficacy of once daily oral 4′-FIU against lethal NiV infection in the hamster model.

PK assessment revealed sustained plasma exposure of the 4’-FlU ribonucleobase and favorable lung and brain tissue exposure of its bioactive anabolite 4’-FIU-TP in Syrian golden hamsters. This tissue selection was driven by disease relevance, since respiratory tract and CNS are preferentially targeted by NiV depending on inoculum amount, route of infection, and duration of disease [42,51]. Consistent with confirmed oral efficacy of 4’-FIU in a q.d. dosing regimen in other rodent and non-rodent viral infection models [23,52], prolonged tissued exposure of 4’-FIU-TP appeared also compatible in Syrian golden hamsters although CNS exposure was significantly lower compared to that obtained in mice despite similar plasma exposure [38]. Accordingly, both 10 and 25 mg/kg dose levels were explored in this study in an attempt to best align exposure levels with those seen in other infection models with confirmed antiviral efficacy [21,29,52].

We opted for a high virus inoculum of 10^4-5^ PFU/animal to ensure uniform lethality in the vehicle control groups, although we are aware that a large inoculum can favor respiratory presentation of NiV disease and shorten the time to death compared to lower challenge amounts [42]. To compensate for this potential respiratory bias, virus was administered IP, which facilitates host invasion beyond the respiratory tract and supports faster dissemination compared to the intranasal route [51].

Reduced viral shedding and lower tissue viral load in all our *in vivo* studies corroborated the outcome of previous work examining efficacy of 5 and 20 mg/kg 4’-FlU in non-lethal models of RSV and SARS-CoV-2 infection in mice, hamsters, and/or ferrets [21,29]. Increasing the length of a 4’-FlU treatment course from 7 to 21/28 days significantly improved survival and decreased the number of animals with detectable virus in tissues, demonstrating strong therapeutic benefit of 4’-FlU against a lethal NiV challenge. In addition, prolonged treatment largely eliminated viral shedding, lowered viremia below detection limit and prevented systemic inflammation. Consistent with this, lung lesions were less severe and frequent, supporting that effective management of NiV infections with 4’-FlU will require a long treatment course. In contrast, short 3-dose treatment courses with 4’-FlU were sufficient for complete survival and full therapeutic benefit in a lethal mouse model of pandemic influenza virus infection [23]. Furthermore, a dose as low as 0.5 mg/kg (QOD, orally) of 4’-FIU provided full protection in a lethal guinea pig arenavirus (JUNV) infection model when treatment was initiated at 7 days post challenge and continued for 12 days [25]. This finding clearly highlights differences of affinity or specificity of 4′-FIU-TP for different viral RNA-dependent RNA polymerases, which is consistent with different MOAs, immediate [21,23] versus delayed [23] chain termination, reported for different viral targets.

By comparison, dosing Syrian golden hamsters with ribavirin (50–60 mg/kg) or 6-aza-uridine (175 mg/kg) daily and beyond the time of disease onset only slightly delayed time to death (1–5 days) after lethal infection with the NiV Malaysia prototypic strain [10,11]. Orally available 4′-FIU offered a significantly longer time of protection and the advantage of being able to cross the BBB, as opposed to ribavirin [53], which makes it compatible with rapid dissemination to outpatients in an outbreak setting. On the other hand, the remdesivir (GS-5734) prodrug protected non-human primates (NHP) when administered for 12-days starting 24 hpi [14]. However, virus was detected in the brain of some animals and intravenous administration to outpatients would not be practical. Favipiravir (T-705), another nucleoside analog that works as a viral mutagen, protected hamsters against NiV Malaysia when treated orally or subcutaneously for 14 days [15]. However, its efficacy was reduced in a recent study using a higher infective dose [16]. Dosing is typically higher than 300 mg/kg and the drug is currently approved only in Japan against influenza viruses [54]. Suboptimal treatment is of particular concern for NiV as it can persist and cause late or relapse encephalitis [55]. To address this, increased antiviral activity of 4′-FIU could be gained, tolerability permitting, by combinational treatments with another analogue such as favipiravir but this will require further investigations. Successful combinatorial treatment of JUNV infection has been previously documented using suboptimal doses of favipiravir and a membrane fusion inhibitor [56].

Clinical resistance to nucleoside or nucleotide analogs has previously been documented during treatment of HIV, human cytomegalovirus, HSV, and HBV infections, and has compromised drug efficacy [57–60]. Comparison of whole genome sequences from vehicle- versus 4’-FlU-experienced virus populations identified three candidate mutations with high allele frequency that may affect viral susceptibility to 4′-FIU. None of these SNVs were found in the brains of untreated NiV-infected controls, suggesting they are unlikely to be associated with tissue-specific adaptation. However, shifts in EC_50_ values after rebuilding of these changes in the genetically controlled background of rNiV-B Gluc were moderate, raising the question of whether continued treatment or a higher dose than 10mg/kg would have overcome this low-level resistance. None of the three mutations provided a clear fitness advantage *in vitro* suggesting that they are less likely to be maintained in future viral populations. It is currently unknown whether these changes modify virulence or transmissibility and this will require further investigations. Interestingly, mutations lowering influenza virus susceptibility to 4’-FlU were previously attenuating or reducing transmissibility and moderate resistance could be overcome *in vivo* through standard or slightly elevated 4’FlU dose levels [61]. Besides, no robust resistance to 4’-FlU emerged when the compound was tested against alphaviruses [62,63], altogether indicating that a high genetic barrier prevents robust viral resistance to 4’-FlU. The influenza virus escape mutations lined the interior cavity of the IAV polymerase complex, potentially allowing accommodation of nascent RNA secondary structure changes after incorporation of 4’-FlU [62]. The NiV N-T191S and L-T1341I mutations are located in close proximity to the encapsidated viral genome and the polymerase PRNTase domain, respectively, and could likewise be involved in increasing tolerance to RNA secondary structure changes after 4’-FlU-TP incorporation [43,44]. In contrast, the I286V substitution in the P protein maps to the N-terminal coiled-coil domain [43,45] and it is unclear how this region may contribute to lowering susceptibility to 4’-FlU. Conceivably, this mutation may affect polymerase processivity, reducing viral susceptibility to 4’-FlU through a secondary mechanism.

While fundamental treatment parameters for use of 4’-FlU against NiV are yet to be determined, our study established antiviral efficacy in a relevant animal model of respiratory and CNS disease. Future work will reveal the optimal length of a treatment course, the therapeutic time window available for initiation of efficacious treatment, and the lowest dose required for full therapeutic benefit.

## Supporting information

S1 Fig *In vitro* efficacy of 4’-FIU against rNiV-B Gluc and NiV-B replication. Determination of half-maximal effective concentration (EC_50_) against rNiV-B Gluc (left panel) and NiV-B (right panel) in Vero at day 2 post-infection using a luminescence reporter or plaque reduction assay, respectively. Values indicate the percentage of virus replication based on intensity of reporter gene expression (left panel) or plaque count (right panel) from 4’-FIU- versus vehicle-treated infected cells. Results are expressed as the average of biological triplicates, and error bars represent standard deviations.
(TIF)

S2 Fig Efficacy of orally dosed 4’-FIU against an intraperitoneal rNiV-B Gluc challenge in the Syrian hamster model (Cohort 1). Five groups of hamsters received virus through the intraperitoneal route and were then either immediately or 8–10 hours later (delayed) treated daily and for 7 days with vehicle, or 10–25 mg/kg 4’-FIU. One additional group was used as negative control. All six groups were further monitored for 21 days to assess (A-D) body weight change starting day 0. Note that some data sets are (B, C, D) aligned for improving visualization of values from each subject over time. The yellow shade (C, D, and legend) indicates days of treatment. Data in B are presented as mean with replicates. Open symbols (B-D) denote animals that survived challenge. (E, F, G) Summary of the number of subjects per group with detectable amount of infectious virus in the lung, liver, or brain. Infectious virus in tissues was determined by plaque assay and limit of detection was set to 10^2^ PFU/gram. Note that the number of subjects analyzed per group vary due to animals also succumbing to infection between time points of collection. One way ANOVA followed by Tukey’s multiple comparisons test was used to compare weight loss, shedding, and viremia data between groups. Fisher’s exact test was used to compare the number of subjects with virus in tissues between two groups. Asterisks (*, **, ****) indicate statistical differences of respectively p < 0.05, 0.01, or 0.0001 between groups.
(TIF)

S3 Fig Effect of prolonged 4’-FIU treatment against an intraperitoneal rNiV-B Gluc challenge in the Syrian hamster model (Cohort 2). Four groups of hamsters (n = 10 per group, male and female evenly distributed) received virus through the intraperitoneal route and were then dose similarly as in cohort 1 for 28 days total or 21 consecutive days with 10 mg/kg 4’-FIU. Two additional groups were used as virus and negative controls. (A, B, C, D) Weight loss was monitored throughout the study starting day 0. Note that some data sets are (A, C, D) aligned for improving visualization of values from each subject over time. The yellow shade (C, D, and legend) indicates days of treatment. (E, left) Seroconversion level of each 4’-FIU-treated animal to infection was determined using a Luminescence Reduction Neutralization Test (LNRT) in Vero, and is expressed as the effective concentration required to reduce the virus replication-related luminescence signal by 50% (LRNT_50_). The horizontal dotted line depicts the 1:10 serum dilution. (E, right) Controls of negative (Mock and Virus+ vehicle groups) and positive (nAH1.3 antibody) neutralization are shown and results are expressed in percentage of virus replication compared to untreated infected cells. Open symbols (B-E) correspond to values from subjects succumbing to disease. One way ANOVA followed by Tukey’s multiple comparisons test was used to compare weight loss data at end of treatments. Unpaired t test was used to compare LRNT_50_ values between the 28- and 21-days treatments. Asterisks (*, **) indicate statistical differences of respectively p < 0.05 and 0.01 between groups. “ns” for non-significant. Data in E are presented as mean from replicates.
(TIF)

S4 Fig Effect of prolonged 4’-FIU treatment on rNiV-B Gluc dissemination in the Syrian hamster model (Cohort 2). (A, B, C, D) Summary of the number of subjects per group with detectable amount of infectious virus in the blood, lung, liver, or brain. Infectious virus in sera and tissues was determined by plaque assay and limit of detection was set to 10^1.82^ PFU/ml or 10^2^ PFU/gram. Note that the number of subjects analyzed per group vary due to animals also succumbing to infection between time points of collection. Fisher’s exact test was used to compare the number of subjects with virus in sera or tissues between two groups. Asterisks (*, **, ***, ****) indicate statistical differences of respectively p < 0.05, 0.01, 0.001, or 0.0001 between groups.
(TIF)

S5 Fig Systemic inflammatory response of rNiV-B Gluc-infected Syrian hamsters, and effect of prolonged 4’-FIU treatment. Concentrations of cytokines in sera of mock- and virus-infected Syrian hamsters at the end of study or peak disease prior euthanasia. Individual values are shown along with mean and standard deviation for each group. Open symbols correspond to values from subjects succumbing to disease. Asterisks (*, ****) indicate statistical differences of respectively p < 0.05, or 0.0001 between groups. “ns” for not significant.
(TIF)

S6 Fig RAVA analysis showing frequency of mutations (synonymous/nonsynonymous/complex) per viral protein and tissue when comparing virus in tissues to virus stock. Threshold: allele frequency ≥1%, coverage depth ≥2. Note that the SNV highlighted in red (square symbol) on L was also found in tissues from the virus only group.
(TIF)

S1 Table Clinical signs of disease following rNiV-B Gluc intraperitoneal challenge (cohort 2). (DOCX)

S2 Table Histopathology of lung and brain sections from the cohort 2 study. Cells shaded in grey correspond to animals that succumbed to disease or met euthanasia criteria prior end of study. Lung histology score: (0) no remarkable lesions, (A1) mild increase in inflammatory cells in the perivascular region and/or interstitium, (A2) infiltration of inflammatory cells, primarily neutrophils in the perivascular region and/or interstitium, (A3) nodular lesions mainly consisting of mononuclear cells in the perivascular region and/or interstitium, (A4) necrotic and/or hemorrhagic lesions in the perivascular region and/or interstitium, (B1) infrequent lung edema and/or fibrinous exudates, (B2) lung edema and/or fibrinous exudate mainly associated with interstitium lesions, (B3) widespread lung edema and/or fibrinous exudates, (C1) infrequent formation of syncytia in the vascular endothelium, (C2) frequent formation of syncytia in the vascular endothelium. Brain histology score: (0) no remarkable lesions, (A1) mild increase in mononuclear cells in the perivascular region, (A2) moderate perivascular cuffing with mononuclear cells, (B1) mild meningitis, (B2) moderate meningitis, (C1) focal demyelination, (C2) focal necrosis. Virus genome: (-) negative/under limit of detection by RT-qPCR, (+) positive for virus.
(DOCX)

S3 Table Reference based Analysis of Viral Alleles (RAVA) analysis recapitulating amino acid changes and corresponding frequency between the reference virus sequence (AY988601) incorporating Gaussia luciferase and eGFP reporter genes, and the virus stock used for animal challenges. Threshold: allele frequency ≥25%, coverage depth ≥30.
(DOCX)

S4 Table Sequencing raw data recapitulating the total number of reads per tissue sample and mean coverage nucleotide using virus stock as reference sequence. (DOCX)

S5 Table Reference based Analysis of Viral Alleles (RAVA) analysis recapitulating nonsynonymous and stopgain mutations that are solely found in virus isolated from 4’-FIU-treated animals. Threshold: allele frequency ≥25%, coverage depth ≥30. The asterisk (*) represents a stop codon.
(DOCX)

## References

[1] Singh RK, Dhama K, Chakraborty S, Tiwari R, Natesan S, Khandia R, et al. Nipah virus: epidemiology, pathology, immunobiology and advances in diagnosis, vaccine designing and control strategies - a comprehensive review. Vet Q. 2019;39(1):26–55. doi: 10.1080/01652176.2019.1580827 31006350 PMC6830995

[2] Ukoaka BM, Okesanya OJ, Daniel FM, Ahmed MM, Udam NG, Wagwula PM, et al. Updated WHO list of emerging pathogens for a potential future pandemic: Implications for public health and global preparedness. Infez Med. 2024;32(4):463–77. doi: 10.53854/liim-3204-5 39660154 PMC11627490

[3] Aditi, Shariff M. Nipah virus infection: A review. Epidemiol Infect. 2019;147:e95. doi: 10.1017/S0950268819000086 30869046 PMC6518547

[4] Goh KJ, Tan CT, Chew NK, Tan PS, Kamarulzaman A, Sarji SA, et al. Clinical features of Nipah virus encephalitis among pig farmers in Malaysia. N Engl J Med. 2000;342(17):1229–35. doi: 10.1056/NEJM200004273421701 10781618

[5] Sejvar JJ, Hossain J, Saha SK, Gurley ES, Banu S, Hamadani JD, et al. Long-term neurological and functional outcome in Nipah virus infection. Ann Neurol. 2007;62(3):235–42. doi: 10.1002/ana.21178 17696217

[6] Shete AM, Radhakrishnan C, Pardeshi PG, Yadav PD, Jain R, Sahay RR, et al. Antibody response in symptomatic & asymptomatic Nipah virus cases from Kerala, India. Indian J Med Res. 2021;154(3):533–5. doi: 10.4103/ijmr.IJMR_4388_20 35142653 PMC9131784

[7] Gómez Román R, Tornieporth N, Cherian NG, Shurtleff AC, L’Azou Jackson M, Yeskey D, et al. Medical countermeasures against henipaviruses: a review and public health perspective. Lancet Infect Dis. 2022;22(1):e13–27. doi: 10.1016/S1473-3099(21)00400-X 34735799 PMC8694750

[8] Rodrigue V, Gravagna K, Yao J, Nafade V, Basta NE. Current progress towards prevention of Nipah and Hendra disease in humans: A scoping review of vaccine and monoclonal antibody candidates being evaluated in clinical trials. Trop Med Int Health. 2024;29(5):354–64. doi: 10.1111/tmi.13979 38415314

[9] Chan XHS, Haeusler IL, Choy BJK, Hassan MZ, Takata J, Hurst TP, et al. Therapeutics for Nipah virus disease: a systematic review to support prioritisation of drug candidates for clinical trials. Lancet Microbe. 2025;6(5):101002. doi: 10.1016/j.lanmic.2024.101002 39549708 PMC12062192

[10] Freiberg AN, Worthy MN, Lee B, Holbrook MR. Combined chloroquine and ribavirin treatment does not prevent death in a hamster model of Nipah and Hendra virus infection. J Gen Virol. 2010;91(Pt 3):765–72. doi: 10.1099/vir.0.017269-0 19889926 PMC2888097

[11] Georges-Courbot MC, Contamin H, Faure C, Loth P, Baize S, Leyssen P, et al. Poly(I)-poly(C12U) but not ribavirin prevents death in a hamster model of Nipah virus infection. Antimicrob Agents Chemother. 2006;50(5):1768–72. doi: 10.1128/AAC.50.5.1768-1772.2006 16641448 PMC1472238

[12] Rockx B, Bossart KN, Feldmann F, Geisbert JB, Hickey AC, Brining D, et al. A novel model of lethal Hendra virus infection in African green monkeys and the effectiveness of ribavirin treatment. J Virol. 2010;84(19):9831–9. doi: 10.1128/JVI.01163-10 20660198 PMC2937751

[13] de Wit E, Williamson BN, Feldmann F, Goldin K, Lo MK, Okumura A, et al. Late remdesivir treatment initiation partially protects African green monkeys from lethal Nipah virus infection. Antiviral Res. 2023;216:105658. doi: 10.1016/j.antiviral.2023.105658 37356729 PMC10529221

[14] Lo MK, Feldmann F, Gary JM, Jordan R, Bannister R, Cronin J, et al. Remdesivir (GS-5734) protects African green monkeys from Nipah virus challenge. Sci Transl Med. 2019;11(494):eaau9242. doi: 10.1126/scitranslmed.aau9242 31142680 PMC6732787

[15] Dawes BE, Kalveram B, Ikegami T, Juelich T, Smith JK, Zhang L, et al. Favipiravir (T-705) protects against Nipah virus infection in the hamster model. Sci Rep. 2018;8:7604. doi: 10.1038/s41598-018-25780-3 29765101 PMC5954062

[16] Johnston SC, Qiu J, Norris SLW, Panchal R, Punger EM, Teague M, et al. Dose response comparison of Nipah virus strains Malaysia and Bangladesh in hamsters exposed by the intranasal or intraperitoneal route. PLoS One. 2025;20(5):e0318912. doi: 10.1371/journal.pone.0318912 40354368 PMC12068590

[17] Hotard AL, He B, Nichol ST, Spiropoulou CF, Lo MK. 4’-Azidocytidine (R1479) inhibits henipaviruses and other paramyxoviruses with high potency. Antiviral Res. 2017;144:147–52. doi: 10.1016/j.antiviral.2017.06.011 28629988 PMC5648002

[18] Lo MK, Jordan PC, Stevens S, Tam Y, Deval J, Nichol ST, et al. Susceptibility of paramyxoviruses and filoviruses to inhibition by 2’-monofluoro- and 2’-difluoro-4’-azidocytidine analogs. Antiviral Res. 2018;153:101–13. doi: 10.1016/j.antiviral.2018.03.009 29601894 PMC6066796

[19] Lo MK, Shrivastava-Ranjan P, Chatterjee P, Flint M, Beadle JR, Valiaeva N, et al. Broad-Spectrum In Vitro Antiviral Activity of ODBG-P-RVn: An Orally-Available, Lipid-Modified Monophosphate Prodrug of Remdesivir Parent Nucleoside (GS-441524). Microbiol Spectr. 2021;9:e0153721. doi: 10.1128/Spectrum.01537-21 PMC8612139 34817209

[20] McMillan RE, Lo MK, Zhang XQ, Beadle JR, Valiaeva N, Garretson AF, et al. Enhanced broad spectrum in vitro antiviral efficacy of 3-F-4-MeO-Bn, 3-CN, and 4-CN derivatives of lipid remdesivir nucleoside monophosphate prodrugs. Antiviral Res. 2023;219:105718. doi: 10.1016/j.antiviral.2023.105718 37758067 PMC10790242

[21] Sourimant J, Lieber CM, Aggarwal M, Cox RM, Wolf JD, Yoon J-J, et al. 4’-Fluorouridine is an oral antiviral that blocks respiratory syncytial virus and SARS-CoV-2 replication. Science. 2022;375(6577):161–7. doi: 10.1126/science.abj5508 34855509 PMC9206510

[22] Chen Y, Li X, Han F, Ji B, Li Y, Yan J, et al. The nucleoside analog 4’-fluorouridine suppresses the replication of multiple enteroviruses by targeting 3D polymerase. Antimicrob Agents Chemother. 2024;68(6):e0005424. doi: 10.1128/aac.00054-24 38687016 PMC11620493

[23] Lieber CM, Aggarwal M, Yoon J-J, Cox RM, Kang H-J, Sourimant J, et al. 4’-Fluorouridine mitigates lethal infection with pandemic human and highly pathogenic avian influenza viruses. PLoS Pathog. 2023;19(4):e1011342. doi: 10.1371/journal.ppat.1011342 37068076 PMC10138230

[24] Yin P, May NA, Lello LS, Fayed A, Parks MG, Drobish AM, et al. 4’-Fluorouridine inhibits alphavirus replication and infection in vitro and in vivo. mBio. 2024;15(6):e0042024. doi: 10.1128/mbio.00420-24 38700353 PMC11237586

[25] Welch SR, Spengler JR, Westover JB, Bailey KW, Davies KA, Aida-Ficken V, et al. Delayed low-dose oral administration of 4’-fluorouridine inhibits pathogenic arenaviruses in animal models of lethal disease. Sci Transl Med. 2024;16(774):eado7034. doi: 10.1126/scitranslmed.ado7034 39565871 PMC11875022

[26] Westover JB a J, Kie Hoon, Bailey KW, Kolykhalov AA, Bluemling GR, Natchus MG, et al. Oral 4’-fluorouridine rescues mice from advanced lymphocytic choriomeningitis virus infection. SSRN. 2024. doi: http://dx.doi.org/10.2139/ssrn.5026896 10.1016/j.antiviral.2025.106122 PMC12009201 39993450

[27] Cross RW, Turcinovic J, Prasad AN, Borisevich V, Agans KN, Deer DJ, et al. Oral 4’-fluorouridine rescues nonhuman primates from advanced Lassa fever. Nature. 2026. doi: 10.1038/s41586-025-09906-y PMC12935548 41501462

[28] Haas GD, Kowdle S, Schmitz KS, Azarm KD, Johnson KN, Klain WR, et al. Tetracistronic minigenomes elucidate a functional promoter for Ghana virus and unveils Cedar virus replicase promiscuity for all henipaviruses. J Virol. 2024;98(10):e0080624. doi: 10.1128/jvi.00806-24 39345144 PMC11495047

[29] Schrell L, Fuchs HL, Dickmanns A, Scheibner D, Olejnik J, Hume AJ, et al. Inhibitors of dihydroorotate dehydrogenase synergize with the broad antiviral activity of 4’-fluorouridine. Antiviral Res. 2025;233:106046. doi: 10.1016/j.antiviral.2024.106046 39638153

[30] Zhang Y, Yao Y, Song S, Gao G, Peng Y, Liu H, et al. The oral nucleoside drug VV116 is a promising candidate for treating Nipah virus infection. Emerg Microbes Infect. 2025;14(1):2587983. doi: 10.1080/22221751.2025.2587983 41257471 PMC12632212

[31] Harcourt BH, Lowe L, Tamin A, Liu X, Bankamp B, Bowden N, et al. Genetic characterization of Nipah virus, Bangladesh, 2004. Emerg Infect Dis. 2005;11(10):1594–7. doi: 10.3201/eid1110.050513 16318702 PMC3366751

[32] Yun T, Park A, Hill TE, Pernet O, Beaty SM, Juelich TL, et al. Efficient reverse genetics reveals genetic determinants of budding and fusogenic differences between Nipah and Hendra viruses and enables real-time monitoring of viral spread in small animal models of henipavirus infection. J Virol. 2015;89(2):1242–53. doi: 10.1128/JVI.02583-14 25392218 PMC4300668

[33] Stevens CS, Lowry J, Juelich T, Atkins C, Johnson K, Smith JK, et al. Nipah Virus Bangladesh Infection Elicits Organ-Specific Innate and Inflammatory Responses in the Marmoset Model. J Infect Dis. 2023;228(5):604–14. doi: 10.1093/infdis/jiad053 36869692 PMC10469344

[34] Leon AJ, Borisevich V, Boroumand N, Seymour R, Nusbaum R, Escaffre O, et al. Host gene expression profiles in ferrets infected with genetically distinct henipavirus strains. PLoS Negl Trop Dis. 2018;12(3):e0006343. doi: 10.1371/journal.pntd.0006343 29538374 PMC5868854

[35] Chua KB, Bellini WJ, Rota PA, Harcourt BH, Tamin A, Lam SK, et al. Nipah virus: a recently emergent deadly paramyxovirus. Science. 2000;288(5470):1432–5. doi: 10.1126/science.288.5470.1432 10827955

[36] Chen S, Zhou Y, Chen Y, Gu J. fastp: an ultra-fast all-in-one FASTQ preprocessor. Bioinformatics. 2018;34(17):i884–90. doi: 10.1093/bioinformatics/bty560 30423086 PMC6129281

[37] Lin MJ, Shean RC, Makhsous N, Greninger AL. LAVA: a streamlined visualization tool for longitudinal analysis of viral alleles. bioRxiv. 2019. doi: 10.1101/2019.12.17.879320

[38] Lieber CM, Kang HJ, Sobolik EB, Sticher ZM, Ngo VL, Gewirtz AT, et al. Efficacy of late-onset antiviral treatment in immune-compromised hosts with persistent SARS-CoV-2 infection. bioRxiv. 2024. doi: 10.1101/2024.05.23.595478 PMC11406939 39207133

[39] Pernet O, Schneider BS, Beaty SM, LeBreton M, Yun TE, Park A, et al. Evidence for henipavirus spillover into human populations in Africa. Nat Commun. 2014;5:5342. doi: 10.1038/ncomms6342 25405640 PMC4237230

[40] Rodriguez JJ, Parisien J-P, Horvath CM. Nipah virus V protein evades alpha and gamma interferons by preventing STAT1 and STAT2 activation and nuclear accumulation. J Virol. 2002;76(22):11476–83. doi: 10.1128/jvi.76.22.11476-11483.2002 12388709 PMC136769

[41] Becker N, Maisner A. Nipah Virus Impairs Autocrine IFN Signaling by Sequestering STAT1 and STAT2 into Inclusion Bodies. Viruses. 2023;15(2):554. doi: 10.3390/v15020554 36851768 PMC9967463

[42] Rockx B, Brining D, Kramer J, Callison J, Ebihara H, Mansfield K, et al. Clinical outcome of henipavirus infection in hamsters is determined by the route and dose of infection. J Virol. 2011;85(15):7658–71. doi: 10.1128/JVI.00473-11 21593160 PMC3147900

[43] Yang G, Wang D, Liu B. Structure of the Nipah virus polymerase phosphoprotein complex. Nat Commun. 2024;15(1):8673. doi: 10.1038/s41467-024-52701-y 39375338 PMC11458586

[44] Ker D-S, Jenkins HT, Greive SJ, Antson AA. CryoEM structure of the Nipah virus nucleocapsid assembly. PLoS Pathog. 2021;17(7):e1009740. doi: 10.1371/journal.ppat.1009740 34270629 PMC8318291

[45] Jensen MR, Yabukarski F, Communie G, Condamine E, Mas C, Volchkova V, et al. Structural Description of the Nipah Virus Phosphoprotein and Its Interaction with STAT1. Biophys J. 2020;118(10):2470–88. doi: 10.1016/j.bpj.2020.04.010 32348724 PMC7231922

[46] Weingartl H, Czub S, Copps J, Berhane Y, Middleton D, Marszal P, et al. Invasion of the central nervous system in a porcine host by nipah virus. J Virol. 2005;79(12):7528–34. doi: 10.1128/JVI.79.12.7528-7534.2005 15919907 PMC1143674

[47] Munster VJ, Prescott JB, Bushmaker T, Long D, Rosenke R, Thomas T, et al. Rapid Nipah virus entry into the central nervous system of hamsters via the olfactory route. Sci Rep. 2012;2:736. doi: 10.1038/srep00736 23071900 PMC3471094

[48] Liu J, Coffin KM, Johnston SC, Babka AM, Bell TM, Long SY, et al. Nipah virus persists in the brains of nonhuman primate survivors. JCI Insight. 2019;4(14):e129629. doi: 10.1172/jci.insight.129629 31341108 PMC6675545

[49] Goldin K, Liu Y, Rosenke R, Prado-Smith J, Flagg M, de Wit E. Nipah Virus-Associated Neuropathology in African Green Monkeys During Acute Disease and Convalescence. J Infect Dis. 2025;231(1):219–29. doi: 10.1093/infdis/jiae300 38842160 PMC11793039

[50] Worwa G, Yú S, Hischak AMW, Tran JP, Bearss JJ, Bernbaum J, et al. Human organoids for Risk Group 4 virus research: a new frontier in investigating Nipah virus infection of the central nervous system. J Virol. 2025;99(11):e0107025. doi: 10.1128/jvi.01070-25 41159751 PMC12645973

[51] Findlay-Wilson S, Flett L, Salguero FJ, Ruedas-Torres I, Fotheringham S, Easterbrook L, et al. Establishment of a Nipah Virus Disease Model in Hamsters, including a Comparison of Intranasal and Intraperitoneal Routes of Challenge. Pathogens. 2023;12(8):976. doi: 10.3390/pathogens12080976 37623936 PMC10458503

[52] Lieber CM, Kang H-J, Sobolik EB, Sticher ZM, Ngo VL, Gewirtz AT, et al. Efficacy of late-onset antiviral treatment in immunocompromised hosts with persistent SARS-CoV-2 infection. J Virol. 2024;98(9):e0090524. doi: 10.1128/jvi.00905-24 39207133 PMC11406939

[53] Colombo G, Lorenzini L, Zironi E, Galligioni V, Sonvico F, Balducci AG, et al. Brain distribution of ribavirin after intranasal administration. Antiviral Res. 2011;92(3):408–14. doi: 10.1016/j.antiviral.2011.09.012 22001322

[54] Shiraki K, Daikoku T. Favipiravir, an anti-influenza drug against life-threatening RNA virus infections. Pharmacol Ther. 2020;209:107512. doi: 10.1016/j.pharmthera.2020.107512 32097670 PMC7102570

[55] Tan CT, Goh KJ, Wong KT, Sarji SA, Chua KB, Chew NK, et al. Relapsed and late-onset Nipah encephalitis. Ann Neurol. 2002;51(6):703–8. doi: 10.1002/ana.10212 12112075

[56] Westover JB, Bailey KW, Wasson SR, Boardman KM, Lustig KH, Amberg SM, et al. Coadministration of LHF-535 and favipiravir protects against experimental Junín virus infection and disease. Antiviral Res. 2024;229:105952. doi: 10.1016/j.antiviral.2024.105952 38945484 PMC11323185

[57] Chen H, Lawler JL, Filman DJ, Hogle JM, Coen DM. Resistance to a Nucleoside Analog Antiviral Drug from More Rapid Extension of Drug-Containing Primers. mBio. 2021;12(1):e03492–20. doi: 10.1128/mBio.03492-20 33563814 PMC7885103

[58] Menéndez-Arias L. Mechanisms of resistance to nucleoside analogue inhibitors of HIV-1 reverse transcriptase. Virus Res. 2008;134(1–2):124–46. doi: 10.1016/j.virusres.2007.12.015 18272247

[59] Piret J, Boivin G. Resistance of herpes simplex viruses to nucleoside analogues: mechanisms, prevalence, and management. Antimicrob Agents Chemother. 2011;55(2):459–72. doi: 10.1128/AAC.00615-10 21078929 PMC3028810

[60] Guo X, Wu J, Wei F, Ouyang Y, Li Q, Liu K, et al. Trends in hepatitis B virus resistance to nucleoside/nucleotide analogues in North China from 2009-2016: A retrospective study. Int J Antimicrob Agents. 2018;52(2):201–9. doi: 10.1016/j.ijantimicag.2018.04.002 29654894

[61] Lieber CM, Kang H-J, Aggarwal M, Lieberman NA, Sobolik EB, Yoon J-J, et al. Influenza A virus resistance to 4’-fluorouridine coincides with viral attenuation in vitro and in vivo. PLoS Pathog. 2024;20(2):e1011993. doi: 10.1371/journal.ppat.1011993 38300953 PMC10863857

[62] Lieber CM, Kang HJ, Aggarwal M, Lieberman NA, Sobolik EB, Yoon JJ, et al. Influenza A virus resistance to 4’-fluorouridine coincides with viral attenuation in vitro and in vivo. bioRxiv. 2023. doi: 10.1101/2023.10.20.563370 PMC10863857 38300953

[63] Yin P, Sobolik EB, May NA, Wang S, Fayed A, Vyshenska D, et al. Mutations in chikungunya virus nsP4 decrease viral fitness and sensitivity to the broad-spectrum antiviral 4’-Fluorouridine. PLoS Pathog. 2025;21(1):e1012859. doi: 10.1371/journal.ppat.1012859 39804924 PMC11759387

