# Supplementary material for: Efficacy of the nucleoside analog 4′-Fluorouridine against Nipah virus in the Syrian hamster model
Source: PLoS Pathog. 2026 Apr 3;22(4):e1014093. doi: 10.1371/journal.ppat.1014093 (PMC13048487; doi:10.1371/journal.ppat.1014093)
Supplement: S1 Table — (DOCX) [file ppat.1014093.s007.docx]

| Groups  Symptoms | Virus | Virus + 4’-FIU for 28 days | Virus + 4’-FIU for 21 days |
| --- | --- | --- | --- |
| Neurological signs of disease | 1/10 | 6/20 | 3/20 |
| Respiratory signs of disease | 6/10 | 0/20 | 5/20 |
