# Supplementary material for: Efficacy of the nucleoside analog 4′-Fluorouridine against Nipah virus in the Syrian hamster model
Source: PLoS Pathog. 2026 Apr 3;22(4):e1014093. doi: 10.1371/journal.ppat.1014093 (PMC13048487; doi:10.1371/journal.ppat.1014093)
Supplement: S2 Table — Cells shaded in grey correspond to animals that succumbed to disease or met euthanasia criteria prior end of study. Lung histology score: (0) no remarkable lesions, (A1) mild increase in inflammatory cells in the perivascular region and/or interstitium, (A2) infiltration of inflammatory cells, primarily neutrophils in the perivascular region and/or interstitium, (A3) nodular lesions mainly consisting of mononuclear cells in the perivascular region and/or interstitium, (A4) necrotic and/or hemorrhagic lesions in the perivascular region and/or interstitium, (B1) infrequent lung edema and/or fibrinous exudates, (B2) lung edema and/or fibrinous exudate mainly associated with interstitium lesions, (B3) widespread lung edema and/or fibrinous exudates, (C1) infrequent formation of syncytia in the vascular endothelium, (C2) frequent formation of syncytia in the vascular endothelium. Brain histology score: (0) no remarkable lesions, (A1) mild increase in mononuclear cells in the perivascular region, (A2) moderate perivascular cuffing with mononuclear cells, (B1) mild meningitis, (B2) moderate meningitis, (C1) focal demyelination, (C2) focal necrosis. Virus genome: (-) negative/under limit of detection by RT-qPCR, (+) positive for virus. (DOCX) [file ppat.1014093.s008.docx]

|  |  |  |  | Lung | | Brain | |
| --- | --- | --- | --- | --- | --- | --- | --- |
|  | Inoculum | Treatment | Animal | Histopathology Score | Virus RNA | Histopathology Score | Virus  RNA |
|  | Mock | Vehicle  (Female) | 1 | 0 | - | 0 | - |
|  |  |  | 2 | 0 | - | 0 | - |
|  |  |  | 3 | 0 | - | 0 | - |
|  | Virus | Vehicle  (Female) | 11 | A4, B1, C2 | + | 0 | + |
|  |  |  | 13 | A3, A4, B3, C2 | + | C1 (thalamus) | + |
|  |  |  | 14 | A3, A4, B2, C2 | + | 0 | + |
|  |  | Vehicle  (Male) | 16 | A3, A4, B2, C1 | + | 0 | + |
|  |  |  | 17 | A3, A4, B2, C1 | + | 0 | + |
|  |  |  | 18 | A3, A4, B2, C2 | + | C1 (thalamus) | + |
|  |  |  | 19 | A4, B2, C2 | + | 0 | + |
| 28 days dosing | Virus | 4’-FIU  immediate  (Female) | 21 | 0 | - | 0 | - |
|  |  |  | 22 | 0 | - | 0 | - |
|  |  |  | 23 | 0 | - | B1 (cortex) | + |
|  |  |  | 24 | A2, A4, B2 | - | 0 | + |
|  |  |  | 25 | 0 | - | B2 (cortex) | + |
|  |  | 4’-FIU  immediate  (Male) | 26 | 0 | - | 0 | - |
|  |  |  | 27 | 0 | - | 0 | - |
|  |  |  | 28 | 0 | - | 0 | - |
|  |  |  | 29 | 0 | - | 0 | - |
|  |  |  | 30 | 0 | - | 0 | - |
|  | Virus | 4’-FIU  8-10 hrs delay  (Female) | 31 | A1, A2 | - | 0 | + |
|  |  |  | 32 | A1 | - | C1 (thalamus) | - |
|  |  |  | 33 | 0 | - | 0 | - |
|  |  |  | 34 | 0 | - | 0 | - |
|  |  |  | 35 | 0 | - | 0 | + |
|  |  | 4’-FIU  8-10 hrs delay  (Male) | 36 | A1 | - | 0 | - |
|  |  |  | 37 | A1 | - | 0 | - |
|  |  |  | 38 | A1 | - | 0 | - |
|  |  |  | 39 | A1 | - | 0 | - |
|  |  |  | 40 | 0 | - | 0 | - |
| 21 days dosing | Virus | 4’-FIU  immediate  (Female) | 41 | A3 | - | A1 (pons) | + |
|  |  |  | 42 | 0 | - | 0 | - |
|  |  |  | 43 | A1 | - | 0 | - |
|  |  |  | 44 | 0 | - | A1 (cortex) | - |
|  |  |  | 45 | A1 | - | 0 | - |
|  |  | 4’-FIU  immediate  (Male) | 46 | A1 | - | 0 | - |
|  |  |  | 47 | A3, A4, B3 | + | 0 | + |
|  |  |  | 48 | A1 | - | 0 | - |
|  |  |  | 50 | A3 | - | A2, C2 (thalamus) | + |
|  | Virus | 4’-FIU  8-10 hrs delay  (Female) | 51 | A3, A4, B3, C1 | + | 0 | - |
|  |  |  | 52 | A1 | No sample | 0 | - |
|  |  |  | 53 | A1 | - | 0 | - |
|  |  |  | 54 | 0 | - | 0 | - |
|  |  |  | 55 | 0 | - | 0 | - |
|  |  | 4’-FIU  8-10 hrs delay  (Male) | 56 | A1 | - | 0 | - |
|  |  |  | 57 | A3, A4, B3, C2 | + | 0 | + |
|  |  |  | 58 | A3, A4, B3, C1 | + | 0 | + |
|  |  |  | 59 | 0 | - | A1 (pons) | + |
|  |  |  | 60 | 0 | - | 0 | - |
