# Supplementary material for: Efficacy of the nucleoside analog 4′-Fluorouridine against Nipah virus in the Syrian hamster model
Source: PLoS Pathog. 2026 Apr 3;22(4):e1014093. doi: 10.1371/journal.ppat.1014093 (PMC13048487; doi:10.1371/journal.ppat.1014093)
Supplement: S3 Table — Threshold: allele frequency ≥25%, coverage depth ≥30. (DOCX) [file ppat.1014093.s009.docx]

| Sample | Protein | Amino acid | Nucleotide change | Allele frequency (%) |
| --- | --- | --- | --- | --- |
| Virus Stock | F | S207L | T619C, C620T | 99.78 |
| Virus Stock | F | G252D | G755A | 99.94 |
