# Supplementary material for: Efficacy of the nucleoside analog 4′-Fluorouridine against Nipah virus in the Syrian hamster model
Source: PLoS Pathog. 2026 Apr 3;22(4):e1014093. doi: 10.1371/journal.ppat.1014093 (PMC13048487; doi:10.1371/journal.ppat.1014093)
Supplement: S4 Table — (DOCX) [file ppat.1014093.s010.docx]

| Sample | Group | Sex | Tissue | Reads | Mean coverage | SRA accession | biosample accession |
| --- | --- | --- | --- | --- | --- | --- | --- |
| 11 | Virus | Female | Lung | 15,557,172 | 87.46 | SRR32191899 | SAMN46492342 |
| 13 | Virus | Female | Lung | 28,052,527 | 136.08 | SRR32191921 | SAMN46492340 |
| 14 | Virus | Female | Lung | 7,939,842 | 89.01 | SRR32191897 | SAMN46492344 |
| 17 | Virus | Male | Lung | 16,779,658 | 44.84 | SRR32191893 | SAMN46492348 |
| 18 | Virus | Male | Lung | 19,061,162 | 89.07 | SRR32191895 | SAMN46492346 |
| 19 | Virus | Male | Lung | 6,280,060 | 266.53 | SRR32191917 | SAMN46492352 |
| 51 | Virus + 4'-FIU 10mg/kg  21days (delayed) | Female | Lung | 15,314,078 | 232.65 | SRR32191906 | SAMN46492362 |
| 57 | Virus + 4'-FIU 10mg/kg  21days (delayed) | Male | Lung | 16,095,302 | 123.62 | SRR32191902 | SAMN46492366 |
| 58 | Virus + 4'-FIU 10mg/kg  21days (delayed) | Male | Lung | 17,744,078 | 600.33 | SRR32191904 | SAMN46492364 |
| 11 | Virus | Female | Brain | 1,321,808 | 4,259.31 | SRR32191910 | SAMN46492341 |
| 13 | Virus | Female | Brain | 768,028 | 3,429.50 | SRR32191922 | SAMN46492339 |
| 14 | Virus | Female | Brain | 3,769,426 | 18,289.55 | SRR32191898 | SAMN46492343 |
| 16 | Virus | Male | Brain | 694,749 | 1,116.79 | SRR32191920 | SAMN46492349 |
| 17 | Virus | Male | Brain | 1,585,940 | 5,850.55 | SRR32191894 | SAMN46492347 |
| 18 | Virus | Male | Brain | 2,077,052 | 10,305.06 | SRR32191896 | SAMN46492345 |
| 19 | Virus | Male | Brain | 1,058,228 | 4,082.67 | SRR32191918 | SAMN46492351 |
| 23 | Virus + 4'-FIU 10mg/kg  28days | Female | Brain | 361,828 | 305.96 | SRR32191916 | SAMN46492353 |
| 24 | Virus + 4'-FIU 10mg/kg  28days | Female | Brain | 1,348,376 | 7,989.96 | SRR32191914 | SAMN46492355 |
| 25 | Virus + 4'-FIU 10mg/kg  28days | Female | Brain | 5,018,512 | 29,540.49 | SRR32191915 | SAMN46492354 |
| 35 | Virus + 4'-FIU 10mg/kg  28days (delayed) | Female | Brain | 201,902 | 1,297.65 | SRR32191913 | SAMN46492356 |
| 41 | Virus + 4'-FIU 10mg/kg  21days | Female | Brain | 4,740,708 | 27,582.45 | SRR32191911 | SAMN46492358 |
| 47 | Virus + 4'-FIU 10mg/kg  21days | Male | Brain | 1,003,235 | 4,014.18 | SRR32191908 | SAMN46492360 |
| 50 | Virus + 4'-FIU 10mg/kg  21days | Male | Brain | 2,541,019 | 13,239.15 | SRR32191909 | SAMN46492359 |
| 57 | Virus + 4'-FIU 10mg/kg  21days (delayed) | Male | Brain | 1,556,358 | 7,810.56 | SRR32191903 | SAMN46492365 |
| 58 | Virus + 4'-FIU 10mg/kg  21days (delayed) | Male | Brain | 1,290,435 | 4,450.08 | SRR32191905 | SAMN46492363 |
| 59 | Virus + 4'-FIU 10mg/kg  21days (delayed) | Male | Brain | 2,736,076 | 13,649.54 | SRR32191901 | SAMN46492367 |
