# Supplementary material for: Efficacy of the nucleoside analog 4′-Fluorouridine against Nipah virus in the Syrian hamster model
Source: PLoS Pathog. 2026 Apr 3;22(4):e1014093. doi: 10.1371/journal.ppat.1014093 (PMC13048487; doi:10.1371/journal.ppat.1014093)
Supplement: S5 Table — Threshold: allele frequency ≥25%, coverage depth ≥30. The asterisk (*) represents a stop codon. (DOCX) [file ppat.1014093.s011.docx]

| Sample | Group | Tissue | Protein | Amino acid | Nucleotide change | Allele frequency (%) |
| --- | --- | --- | --- | --- | --- | --- |
| #23 | Virus + 4'-FIU 10mg/kg 28days | Brain | N | T191S | A571T | 96.35 |
| #25 | Virus + 4'-FIU 10mg/kg 28days | Brain | L | T1341I | C4022T | 99.85 |
| #41 | Virus + 4'-FIU 10mg/kg 21days | Brain | P | I286V | A856G | 99.94 |
| #35 | Virus + 4'-FIU 10mg/kg 21days | Brain | P | E162* | G484T | 52.73 |
| #35 | Virus + 4'-FIU 10mg/kg 21days | Brain | P | E171* | G511T | 38.27 |
